# Electronic quantum coherence in glycine molecules probed with ultrashort x-ray pulses in real time

**DOI:** 10.1126/sciadv.abn6848

**Published:** 2022-06-01

**Authors:** David Schwickert, Marco Ruberti, Přemysl Kolorenč, Sergey Usenko, Andreas Przystawik, Karolin Baev, Ivan Baev, Markus Braune, Lars Bocklage, Marie Kristin Czwalinna, Sascha Deinert, Stefan Düsterer, Andreas Hans, Gregor Hartmann, Christian Haunhorst, Marion Kuhlmann, Steffen Palutke, Ralf Röhlsberger, Juliane Rönsch-Schulenburg, Philipp Schmidt, Sven Toleikis, Jens Viefhaus, Michael Martins, André Knie, Detlef Kip, Vitali Averbukh, Jon P. Marangos, Tim Laarmann

**Affiliations:** 1 Deutsches Elektronen-Synchrotron DESY, Notkestr. 85, 22607 Hamburg, Germany.; 2 Department of Physics, Imperial College London, Prince Consort Road, London SW7 2AZ, UK.; 3 Faculty of Mathematics and Physics, Charles University, V Holesovickach 2, 180 00 Praha 8, Czech Republic.; 4 Department of Physics, University of Hamburg, Luruper Chaussee 149, 22761 Hamburg, Germany.; 5 The Hamburg Centre for Ultrafast Imaging CUI, Luruper Chaussee 149, 22761 Hamburg, Germany.; 6 Institute of Physics, University of Kassel, Heinrich-Plett-Str. 40, 34132 Kassel, Germany.; 7 Faculty of Electrical Engineering, Helmut Schmidt University, Holstenhofweg 85, 22043 Hamburg, Germany.; 8 Helmholtz Institute Jena, Fröbelstieg 3, 07743 Jena, Germany.; 9 Helmholtz Centre for Heavy Ion Research (GSI), Planckstr. 1, 64291 Darmstadt, Germany.; 10 Friedrich-Schiller-Universität Jena, Max-Wien-Platz 1, 07743 Jena, Germany.; 11 Helmholtz-Zentrum Berlin für Materialien und Energie, Albert-Einstein-Straße 15, 12489 Berlin, Germany.

## Abstract

Here, we use x-rays to create and probe quantum coherence in the photoionized amino acid glycine. The outgoing photoelectron leaves behind the cation in a coherent superposition of quantum mechanical eigenstates. Delayed x-ray pulses track the induced coherence through resonant x-ray absorption that induces Auger decay and by photoelectron emission from sequential double photoionization. Sinusoidal temporal modulation of the detected signal at early times (0 to 25 fs) is observed in both measurements. Advanced ab initio many-electron simulations allow us to explain the first 25 fs of the detected coherent quantum evolution in terms of the electronic coherence. In the kinematically complete x-ray absorption measurement, we monitor its dynamics for a period of 175 fs and observe an evolving modulation that may implicate the coupling of electronic to vibronic coherence at longer time scales. Our experiment provides a direct support for the existence of long-lived electronic coherence in photoionized biomolecules.

## Teaser

The quantum mechanically evolving electron charge distribution in glycine molecules is monitored via direct real-time measurement.

## INTRODUCTION

Understanding the formation and temporal evolution of electronic coherences and the dynamics of coherent superpositions of many-electron configurations forming electronic wave packets that propagate in space and time is one of the grand challenges in ultrafast science. Electronic coherence may well play a role in photochemical change in matter, particularly in the onset of radiation damage under a wide range of scenarios, from single-molecule x-ray imaging to radiobiology. Its effect in these diverse branches of science, while likely to be present, is yet to be fully understood. Identifying the coherent quantum effects in the radiation damage processes at the molecular level can pave the way to controlling, optimizing, and engineering the ionizing radiation damage similarly to how Auger electron-emitting agents are already used in radiotherapy (*1*), while the much more recently discovered interatomic decay phenomena are projected to play a role in fundamental and applied radiobiology (*2*).

In recent years, intensive debate was stimulated by a seminal study reporting long-lived electronic coherences in polyatomic molecules (*3*). There, extreme ultraviolet (XUV) pulses ionize the amino acid phenylalanine. A coherent superposition involving several electronic states in the cation is formed because of the broad spectral bandwidth of the sub-femtosecond (fs) XUV pulse. Near-infrared pulses probe the initiated coherent dynamics by photofragmentation. The recorded ion fragmentation pattern shows an oscillatory behavior as a function of pump-probe delay with a time period of 4.3 fs that persists for tens of fs. Matching the corresponding energy difference of relevant energy levels, the results have been interpreted as long-lived electronic coherences. Still, the key interrelated questions are whether the electronic coherence can really be that robust and whether it can be detected directly, i.e., through electronic observables. X-ray electron spectroscopy might be regarded as the gold standard of electronic structure–sensitive techniques. Applied in a time-resolved experimental pump-probe scheme, it allows to unambiguously monitor in a kinematically complete experiment the transient electronic coherence, while it evolves long before fragmentation sets in.

The picture of charge migration as purely electronic wave packet dynamics of a coherent superposition of cationic many-electron states (*4*) only holds within the strict Born-Oppenheimer approximation, where one assumes the adiabatic separation between the electronic and the nuclear degrees of freedom to be exact. It is widely appreciated, however, that, especially in polyatomic molecules of relevance to charge migration, the Born-Oppenheimer approximation breaks down spectacularly, for example, because of conical intersections between the adiabatic potential energy surfaces (*5*). This is relevant, for example, for the regions of partial and full breakdown of the molecular orbital picture of ionization (*6*), with narrow energy gaps between multiple electronic states of mixed one-hole (1h) and two-hole one-particle (2h1p) character being on the order of the vibrational quanta. It is, therefore, a relevant open question, to what extent and on what time scale can the original purely electronic picture of charge migration serve as an adequate approximation for the combined electron-nuclear dynamics triggered by molecular ionization? The answer to this question may well be strongly system dependent.

Recently, the quantum dynamics of nuclear wave packets in phenylalanine has been shown to be prone to destroy electronic coherences within just a few fs (*7*). Theoretical works treating nuclear dynamics classically found that electron-hole migration in other prototypical model systems, such as tryptophan (*8*), benzene (*9*), and glycine (*10*), is not substantially perturbed by nuclear motion within the first 20 fs after the excitation. In these calculations, electronic wave packet decoherence partly stems from the nuclear geometry uncertainty related to the zero-point energy (*11*), which is also at the core of our present theoretical treatment and partly from the nuclear wave packet dynamics on the potential energy surfaces of the coherently populated electronic states (*7*). Adding to the controversy, all the experimental evidence on coherence-driven charge migration on the scale of tens of fs available so far relied on the indirect detection channels, e.g., ionic fragment detection (*3*), and no direct measurements (i.e., such as based on the electronic observables) of coherent multielectron dynamics have been available.

In the present contribution, we monitor the birth, propagation, and fate of an electronic wave packet generated in the interaction of fs x-ray pulses with glycine molecules by means of time-resolved electron spectroscopy at the carbon K-edge from a second fs x-ray probe pulse. The second electron emission in the probe process results from two alternative mechanisms that we have isolated in a kinematically complete multiparticle correlation experiment to study them independently of each other despite the many energy absorption and redistribution channels opened up by the soft x-ray pump pulse. First, a resonant transition can be induced from the carbon core shell into the selected ionized valence orbital of interest (x-ray absorption), resulting in the Auger decay of the formed core-hole state. Second, a sequential double ionization of the valence electron shell takes place, a process with weak orbital and spatial selectivity due to the molecular character of the valence orbitals with only moderately orbital-dependent photoionization cross sections, which, nevertheless, carries signatures of the temporal evolution of the electronic states of the cation.

The x-ray absorption process, first proposed as a valence-hole coherence probe in (*12*), involving a localized core orbital and a resonant transition is characterized by high element and orbital selectivity. Thereby, we probe the transient local electron-hole density in inner-valence molecular orbitals directly in the time domain tracing long-lived quantum coherences that result from the initial formation of a coherent superposition of cationic eigenstates in the course of photoionization. Comparison of our experimental results from both channels with high-level ab initio many-electron theory, taking into account the quantum uncertainty in the nuclear geometry, shows that our observations are consistent with the idea of a long-lived electronic coherence.

## RESULTS

**Fig. 1.**
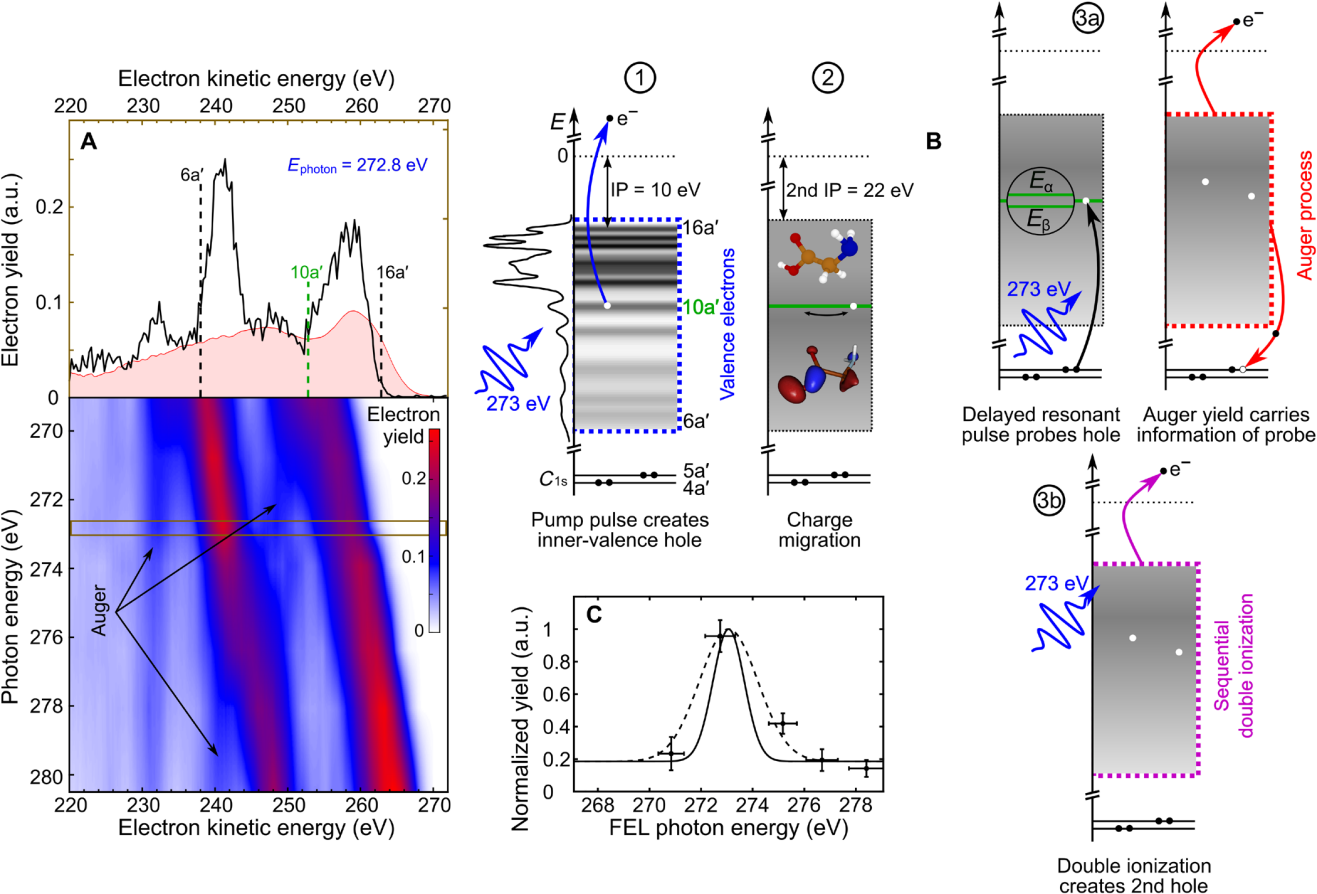
X-ray photoelectron spectroscopy of glycine. (**A**) Kinetic energy distributions of detected electrons as a function of free-electron laser (FEL) photon energy are presented in a false color plot using single pulses, i.e., ∆*t* = 0. Dashed black lines in the upper spectrum recorded at a photon energy of 272.8 eV mark the energy ranges of single photoionization of valence and inner-valence electron orbitals (6a′ to 16a′ and 1a″ to 4a″), respectively. An experimental Auger electron spectrum (shaded red area) for C 1s core vacancies (4a′ and 5a′) is reproduced from (*15*). The center-of-mass kinetic energy corresponding to valence ionization of 10a′ is indicated (dashed green line). (**B**) A single-color pump-probe scheme is applied to track charge dynamics initiated by photoelectron emission from the 10a′ molecular orbital. The total cascade involves several different processes: (1) photoionization, (2) charge migration triggered by coherent population of ionized states during photoionization, (3a) resonant carbon 1s core-hole excitation and Auger decay, and (3b) sequential double photoionization of valence electrons. The individual steps are depicted as a sequence of energy diagrams. The density of states is indicated by using the experimental data reproduced from (*14*). (**C**) Two-electron coincidences plotted as a function of FEL photon energy, with one electron being detected at the kinetic energy corresponding to valence ionization of 10a′. These data are recorded in a second set of measurements with substantially increased data acquisition time by a factor of ~100 compared to (A). The resonance is fitted with a Gaussian envelope (dashed line) and deconvoluted (solid line) with respect to the spectral bandwidth of the FEL pulses (see Fig. 7). The data are normalized to the FEL pulse energy to account for FEL fluctuations and to the maximum signal on resonance (a.u., arbitrary units).

Glycine is the simplest of the 20 natural amino acids acting as molecular building blocks of peptides and proteins. It contains the characteristic amine (─NH_2_) and carboxyl (─COOH) functional groups linked via a methylene bridge and is sketched in the center of Fig. 1B. The neutral molecule has *N* = 40 electrons forming a closed-shell system in its ground state, which can be described by 20 molecular orbitals. A recent theoretical paper reported photoionization cross sections for the different electron orbitals using density functional theory (*13*). The theoretical results describing the static electronic structure are in good agreement with experimental photoelectron spectra recorded at a synchrotron light source (*14*). The valence and inner-valence electron orbitals (6a′ to 16a′ and 1a″ to 4a″) exhibit binding energies *E*_b_ in the range of approximately 10 to 35 eV (*10*, *13*, *14*). The Auger spectrum of glycine resulting from oxygen K-shell ionization has been reported in (*15*).

In our time-resolved experiments, we used a single-color, fs x-ray pump-probe technique (*16*, *17*). The electron dynamics were initiated by free-electron laser (FEL) pulses of a few fs duration at a central photon energy tuned in the range between 269 and 281 eV. Whereas the photon energy was sufficient to ionize electrons from all valence and inner-valence orbitals, the localized 1s core electrons of carbon (~284 eV), nitrogen (~400 eV), and oxygen (~532 eV) were not accessible. The FEL pulses were focused onto the molecular beam produced from glycine powder heated to 160°C. Electrons generated at the intersection volume were detected with a magnetic bottle electron spectrometer. The instrument also allowed for simultaneous measurement of the resulting ion time-of-flight (TOF) spectra. More details on the experimental setup can be found in Materials and Methods.

We measured the kinetic energy *E*_kin_ of emitted electrons as a function of FEL photon energy *E*_FEL_ = *h* · ν tuned below the carbon K-edge (*18*). Figure 1A shows the recorded kinetic energy distributions obtained at zero time delay, comprising contributions from the initial photoelectron emission, the subsequent Auger decay channel opened by the x-ray probe-pulse excitation of localized carbon 1s electrons, and probe-induced photoionization of singly charged glycine cations. Emission bands originating from prompt photoionization of valence and inner-valence orbitals of the neutral molecule with binding energies *E*_b_ shift in their kinetic energy according to *E*_kin_ = *h* · ν − *E*_b_. The overall width of the distribution is ~25 eV in agreement with the published data (*13*, *14*). Additional electron emission bands are observed that do not shift as a function of photon energy. These electrons originate from the probe-induced Auger decay, as their kinetic energy is solely given by the electronic structure of glycine molecules. A competing ionization channel resulting in doubly charged glycine ions is the probe-induced photoemission of valence electrons from the initially formed cation. The low-energy onset of their kinetic energy distribution is shifted toward lower energy by the difference between the first and the second ionization potential (12 eV). Note that the high-energy tail of the kinetic energy distribution may still reach the upper limit, taking into account photoelectron emission from electronically excited cations.

In the following, we discuss the overall coherent dynamics tracked in the present time-resolved experiments. The total cascade involves several different processes: (1) photoionization, (2) charge migration, and (3a) resonant carbon 1s core-hole excitation followed by Auger decay (x-ray absorption channel) or (3b) sequential double photoionization of glycine depicted in Fig. 1B. The resonance-enhanced, probe-induced Auger decay channel (x-ray absorption) becomes clearly visible by plotting the two-electron coincidences as a function of FEL photon energy, with one electron being detected at the kinetic energy corresponding to valence ionization of 10a′. These data are recorded in a second set of measurements with substantially increased data acquisition time by a factor of ~100 and are shown in Fig. 1C.

Upon photoionization with fs x-ray pulses, we generate electronically excited many-body states in the glycine cation (*19*). The FEL spectral bandwidth below 0.45% full width at half maximum (FWHM) was sufficient to form a coherent superposition of several cationic eigenstates. Details on the longitudinal coherence and spectral properties of the applied single-mode FEL pulses can be found in the literature (*20*, *21*) and in Materials and Methods, respectively. The ionization paths leading to the same photoelectron energies, but leaving behind different cationic states, interfere and trigger coherent quantum motion of the remaining *N–1* electrons of the cation. The induced electronic dynamics depend on the involved eigenstates. With the pulse parameters used in the present study, most of the coherence produced in the glycine cation belongs to the ionic eigenstates in the 10a′ band energy region (for details, see section S2.1), i.e., the states resulting from photoelectron emission from the 10a′ orbital with a binding energy of ~20 eV (the 10a′ orbital is visualized in the center of Fig. 1B). Therefore, ionization results in an essentially coherent superposition of a series of cationic eigenstates Ψ*_j_*, each with ~5 to 30% contributions from the inner-valence hole (1h) state and ~70 to 95% of a series of excited 2h1p configurations (*12*). In the latter, an additional bound valence electron is excited owing to electron correlation. The time-dependent electronic wave packet describing the motion of the positive charge created in the photoionization step as a function of time *t* can be written in simple terms as a linear combination

$$\Psi^{N-1}(t)=\sum c_{j}\;e^{-i\frac{E_{j}}{\hbar }t}\;\Psi_{j}$$ (1)

with expansion coefficients *c_j_*. In this picture, the positive charge oscillates with periodicities *T* given by the energy separation of the pairs of involved eigenstates, where ℏ is Planck’s constant over 2π

$$T=\frac{2\pi \hbar }{|E_{i}-E_{j}|}$$ (2)

The *N–1* electron density, integrated over all electron coordinates but one, will also oscillate with the same periodicity because of the constructive and destructive interference of the many-electron states contributing to the wave packet in Eq. 1.

The evolution of the positive charge in the molecular skeleton is followed in this work in two different measurements. On the one hand, we use x-ray absorption, where the transient electron hole states are probed, following a controlled time delay, by excitation of a strongly localized carbon 1s core electron into the “empty” orbital via time-delayed fs x-ray pulses of the same color. As long as the electronic coherence, i.e., the electronic wave packet described in Eq. 1, is preserved, the probability of the resonant x-ray transition (C 1s → 10a′, at 272.7 eV) that fills the inner-valence orbital and produces a carbon 1s core hole depends on the time-dependent amplitudes of the cationic states with a significant 1h component localized on the carbon atom. The corresponding delay-dependent Auger yield is therefore a direct temporal fingerprint of the evolving many-electron wave packet in Eq. 1. Thus, the induced coherent electron dynamics following 10a′ photoionization can be observed directly by recording the subsequent Auger electron emission as a function of x-ray pump–x-ray probe time delay. The basic experimental scheme flanked by ab initio simulations has already been proposed by Cooper *et al.* (*12*) in 2014. The theoretically predicted oscillation period *T* is within 10 to 20 fs according to (*12*, *22*, *23*). On the other hand, we use nonresonant x-ray photoelectron spectroscopy, where the electron dynamics is monitored by photoelectron emission from sequential double photoionization processes. This turns out to be the dominant process (by a factor 10) in the time-resolved measurements at a central photon energy of 274.0 eV (off-resonance), although small contributions from the x-ray probe–induced Auger decay are present in this dataset as well [see Fig. 1 (A and C)].

Here, we will report first the results from the x-ray absorption measurement and, second, the nonresonant sequential double-ionization x-ray spectroscopy channel results. For the former, a higher count rate was achieved, which enabled correlated multiparticle detection to isolate the channel of interest (see Materials and Methods). Otherwise, the conditions in both of these independent measurements are the same.

**Fig. 2.**
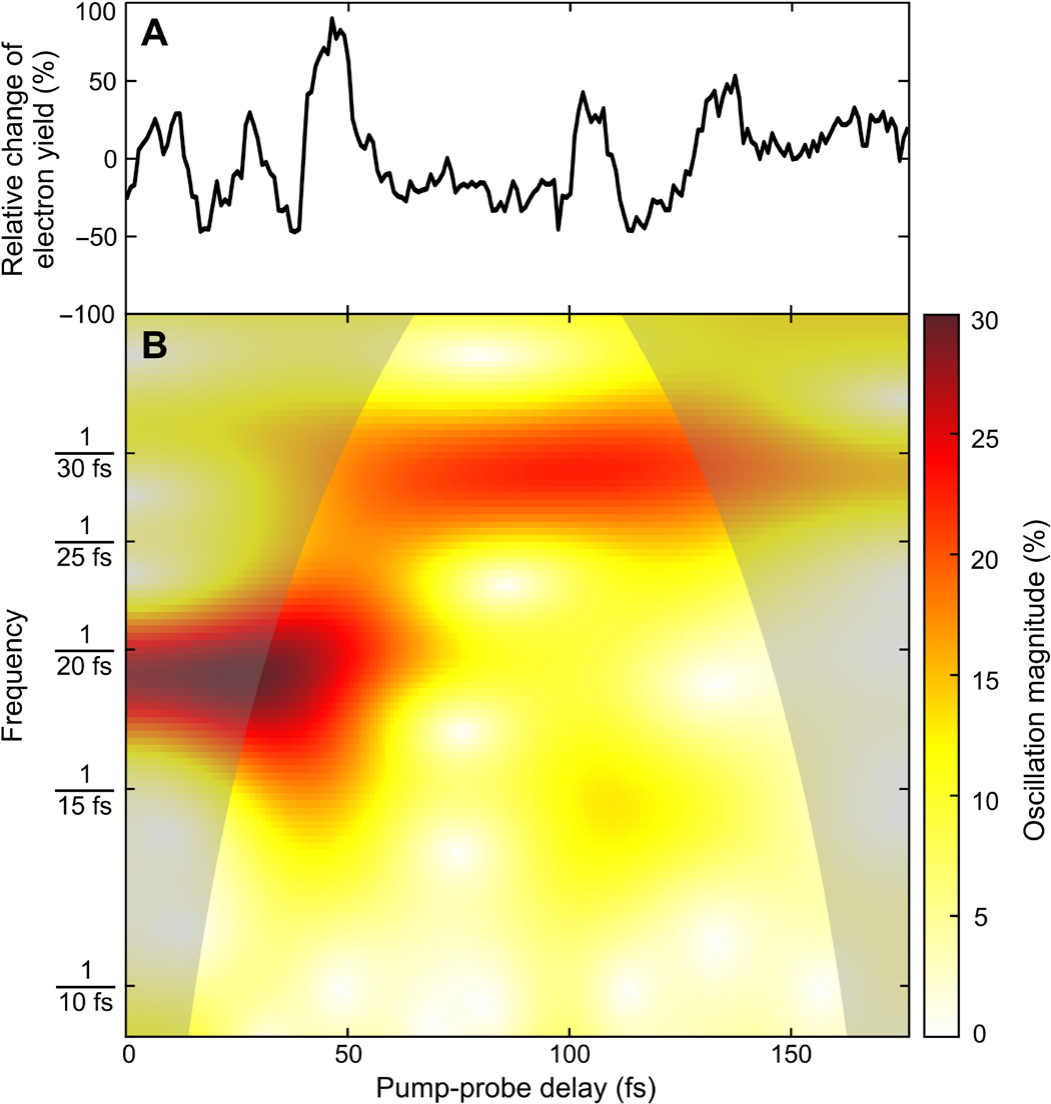
Electron signal oscillations induced and monitored with 272.7-eV photons and wavelet analysis. (**A**) Relative change of the detected electron yield correlated with the generation of a Gly^2+^ parent ion and recorded together with an electron at the kinetic energy corresponding to valence ionization of 10a′ as a function of x-ray pump-probe delay in 1-fs steps (black line). (**B**) The continuous wavelet transform (see Materials and Methods) monitors the magnitude evolution of the nonstationary signal at scaling frequencies (false color plot). The “cone of influence,” where part of the wavelet in time domain extends past the finite recorded experimental signal trace, is given as a darker shaded area. Here, artificial edge effects disturb the frequency analysis, and therefore, peak structures in the shaded area of the color plot are likely underestimated. Two dominant time periods of $T=(19.6_{-1.4}^{+1.5})$ fs (over the first 40 fs) and $T=(29.3_{-2.0}^{+2.2})$ fs (later on) can be extracted, with the initial period being in excellent agreement with the ab initio theory explaining the oscillations as a result of electronic coherence averaged over the nuclear geometries (for details, see the main text).

In the x-ray absorption measurement based on the resonant transition followed by Auger decay probe, we set the FEL photon energy to the resonant x-ray probe transition (C 1s → 10a′) at 272.7 eV, providing spatial selectivity of the element-specific core transition into the spatially extended inner-valence orbital and recorded delay scans over a range of 175 fs. In addition to the resonance effect (see Fig. 1C), this experiment benefited from a substantially higher density of glycine molecules in the focal volume (×100) compared to the experimental campaign probing the nonresonant channel. The increase was achieved by minimizing the distance between the molecular beam pipe and the FEL focus from ~10 to ~1 mm. This allowed us to collect three or more particle events per FEL pulse, comprising the photoelectron at the kinetic energy corresponding to valence ionization of 10a′ under investigation, the final Gly^2+^ parent ion, and pump-probe events including Auger decay. Figure 2A shows the result of this kinematically complete experiment, spanning a 175-fs time scale. We counted the multiparticle events and plotted the relative change of the detected electron yield correlated with the generation of a Gly^2+^ parent ion and recorded together with an electron at the kinetic energy corresponding to valence ionization of 10a′ as a function of x-ray pump-probe delay in 1-fs steps. Oscillations of the detected electron signal in the time domain are clearly visible. The wavelet analysis of the electron signal shown in Fig. 2B reveals the frequency components that exist in the temporal domain (see Materials and Methods for details). The short-time oscillation period was determined as $T\;=(19.6_{-1.4}^{+1.5})$ fs, in excellent agreement with the 20-fs oscillation period predicted by our ab initio many-electron calculations, and exactly matches the period that was observed in the off-resonance sequential double-ionization channel (see below). At resonance, the carbon 1s core-hole excitation channel dominates. The calculations give a ratio between the two processes of 3:1. The sequential process is expected to contribute in minor part to the signal plotted in Fig. 2A. In addition, we observe that the 20-fs-period oscillations in the signal fade away after 40 to 50 fs, and slower oscillations with a time period $T\;=(29.3_{-2.0}^{+2.2})$ fs become dominant and finally dissipate after two to three periods, resulting in the reduced relative change of the detected electron yield for long time delays.

**Fig. 3.**
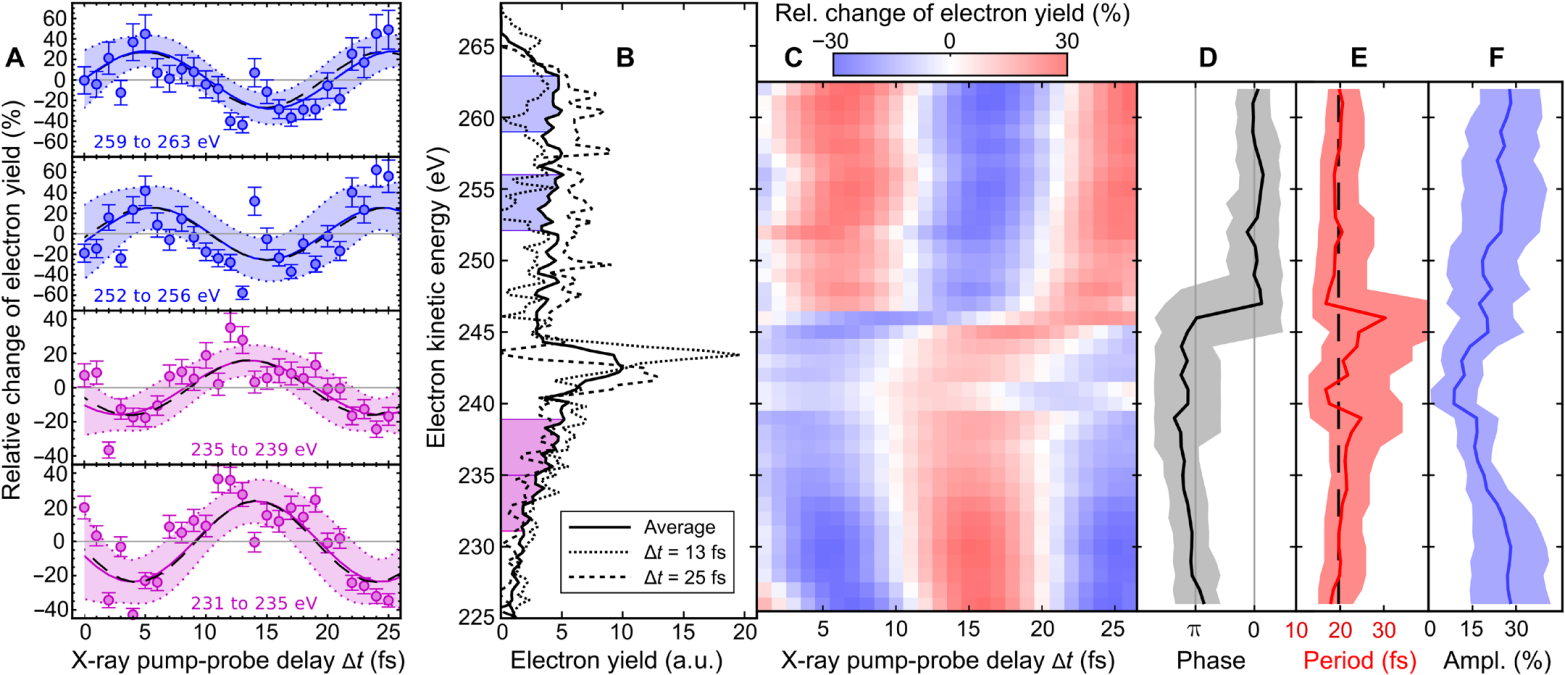
Phase-sensitive electron wave packet interferometry of glycine with 274.0-eV photons. (**A**) Relative change of the detected electron yields in kinetic energy ranges with contributions from Auger emission superimposed with sequential double ionization as a function of x-ray pump-probe delay. The solid lines are the sinusoidal fitting functions of time period *T*. The 95% confidence bounds are given by dotted lines. (**B**) Kinetic energy distributions of detected electrons recorded for pump-probe delays ∆*t* = 13 fs and ∆*t* = 25 fs compared with the average electron spectrum over all delays. (**C**) Delay-dependent electron spectra fitted in steps of 1 eV with sinusoidal functions and 4-eV detection range having the oscillation period, amplitude, and phase as free parameters. The fitting functions are presented as a false color plot. (**D**) Phases, (**E**) periods, and (**F**) amplitudes of the sinusoidal functions (solid black, solid red, and solid blue lines, respectively) depending on the kinetic energy of detected electrons. The 95% confidence bounds are given by the shaded areas. The black dashed lines shown in (A) and (E) are the result of using a single time period $T=(19.6_{-1.4}^{+2.2})$ fs for fitting the complete dataset.

Probing the x-ray–induced electron dynamics off-resonance and, therefore, facing notably lower count rates, Fig. 3A shows the result on the shorter, 25-fs time scale. Sequential double ionization is dominant (by a factor of 10 at least) over the Auger decay for the off-resonance probe FEL photon energy used in this part of our experiment (see fig. S4). Here, we counted the electrons with kinetic energies in four different ranges determined by the FEL spectral bandwidth, (i) from 259 to 263 eV, (ii) from 252 to 256 eV, (iii) from 235 to 239 eV, and (iv) from 231 to 235 eV, and plotted the relative change of the electron yield in each energy bin (i to iv) as a function of pump-probe delay in 1-fs steps. Oscillations of the detected electron signal in the time domain are clearly visible. The curves for high-energy and low-energy electron emission show a π-phase shift with respect to each other, i.e., the electron yield in different energy ranges is maximized/minimized for different x-ray pump-probe delays, as shown in Fig. 3B. To work out for which electron kinetic energy the phase jump occurs, the delay-dependent electron spectra have been fitted in steps of 1 eV, with sinusoidal functions and a 4-eV detection range keeping the oscillation period, amplitude, and phase of the quantum beat as free parameters (see Materials and Methods). The outcome is summarized in Fig. 3 (C to F). We observe the π-phase jump at an electron kinetic energy of (246 ± 2) eV. Above and below this transition, the relative change of the detected electron yield oscillates with a time period of $T=(19.6_{-1.4}^{+2.2})$ fs over the full kinetic energy range of electron emission from 224 to 264 eV shown in Fig. 1A. This time period is in close agreement to the early time oscillations measured in the independent x-ray absorption measurement presented in Fig. 2.

**Fig. 4.**
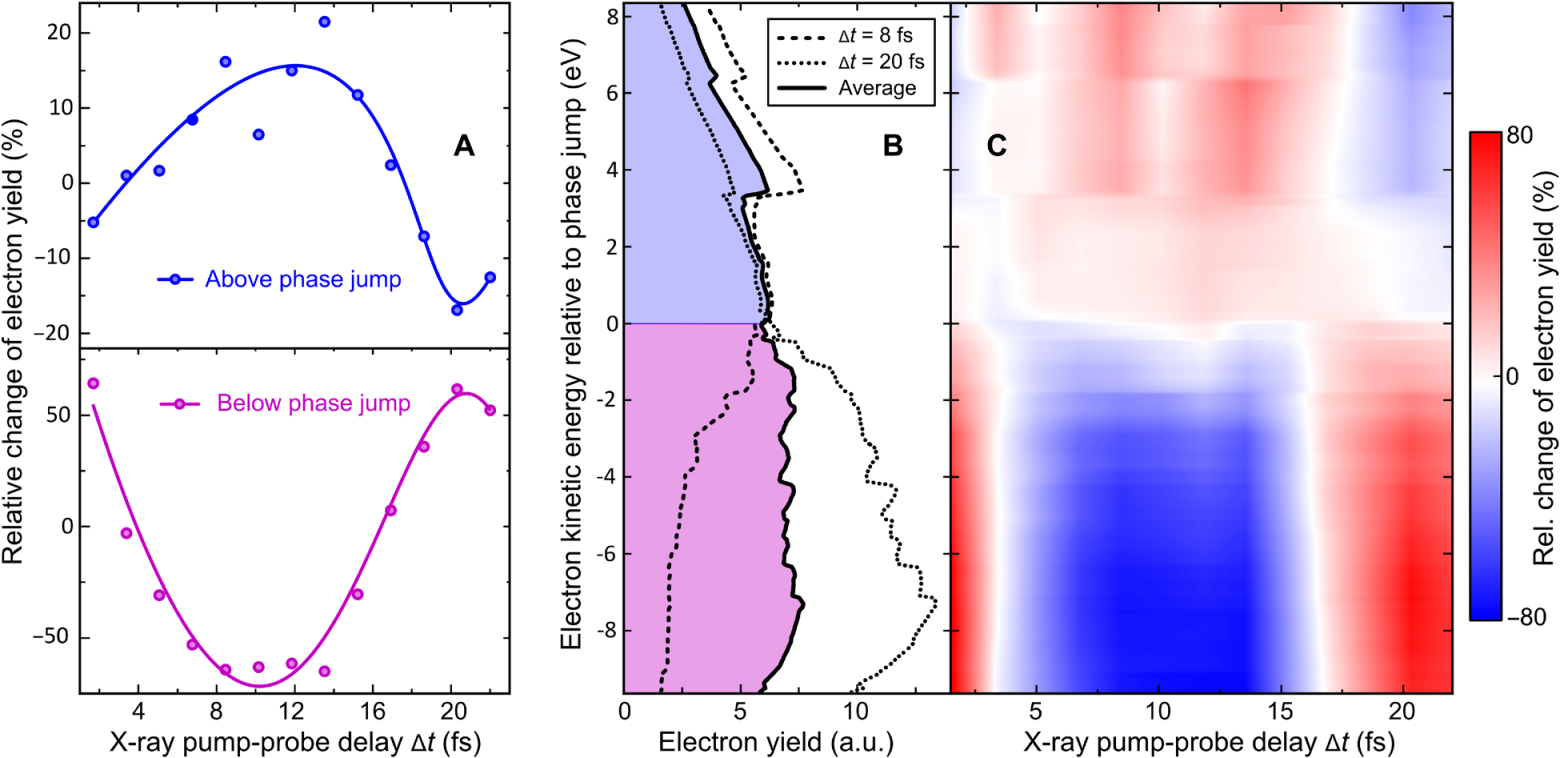
Ab initio time-dependent B-spline restricted correlation space–algebraic diagrammatic construction simulation of experimental observables using the measured FLASH pulse parameters in the off-resonance probe scenario. (**A**) Relative change of the detected electron yields in kinetic energy ranges above and below the phase jump including contributions from Auger emission and sequential double ionization as a function of x-ray pump-probe delay. The solid lines are a guide to the eye, showing the onset of an oscillatory behavior with a time period of ~20 fs. (**B**) Kinetic energy distributions of emitted electrons simulated for pump-probe delays ∆*t* = 8 fs and ∆*t* = 20 fs compared with the average electron spectrum over all calculated delays. (**C**) Delay-dependent electron spectra presented as a false color plot system (for details, see the Supplementary Materials).

We have simulated the experimental observables using the measured free-electron laser in Hamburg (FLASH) pulse parameters and including the various ionization channels. The numerical ab initio results on the probe-induced electron yield modulation in pump-ionized glycine molecules as a function of x-ray pump-probe delay are summarized in Fig. 4 (A to C). Our theoretical simulations show that the phase jump observed in the off-resonance experiment is a property of the sequential double ionization; see further details in section S2.2. Both channels monitor the same beating frequency of the cationic eigenstates.

The calculations were performed using the time-dependent B-spline restricted correlation space–algebraic diagrammatic construction (ADC) simulation method (*24*–*26*), which provided the populations, degrees of coherence, and relative phases between each pair of accessible cationic states computed using the ADC(2,2) method in (*27*), as well as the relative change of electron kinetic energy spectra as a function of pump-probe time delay as recorded in the measurements. The simulation combines the accurate description of the photoelectron continuum by means of the B-spline basis set (*28*) and the ADC description of electron correlation, but in view of the required computational effort that is restricted to a single (equilibrium) geometry of the Gly I conformer. The lack of geometry averaging is probably the main source of discrepancy between the measured (Fig. 3) and simulated (Fig. 4) probe electron spectra. The agreement between both on-resonance and off-resonance experiments and the theory in terms of the initial period of the oscillations of the many-electron wave packet allows us to interpret the initial 25 fs of the quantum evolution of the glycine cation as resulting from the electronic coherence. The amplitude of the observed oscillation reflects the contribution of the 10a′ photoionization channel and the magnitude of the electronic coherence produced within the 10a′ band of many-electron states to the overall signal (see further details in section S2.2 and fig. S3).

The π difference between the oscillation’s phases in the lower and higher kinetic energy ranges of the photoelectron spectrum is due to the different time evolution of the 1h and 2h1p components of the time-dependent ionic state created by the pump pulse in the 10a′ band. The initial decrease of the 10a′ 1h configuration weight is accompanied by a corresponding increase of the 2h1p coefficients. As a result, because the former (the latter) carries most of the oscillator strength for ionization into the highly excited states of the dication with one hole in the 10a′ orbital (into the lowest-energy dicationic states with two holes in outer-valence orbitals), the photoelectron signal in the corresponding lower (higher) kinetic energy range also initially decreases (increases).

**Fig. 5.**
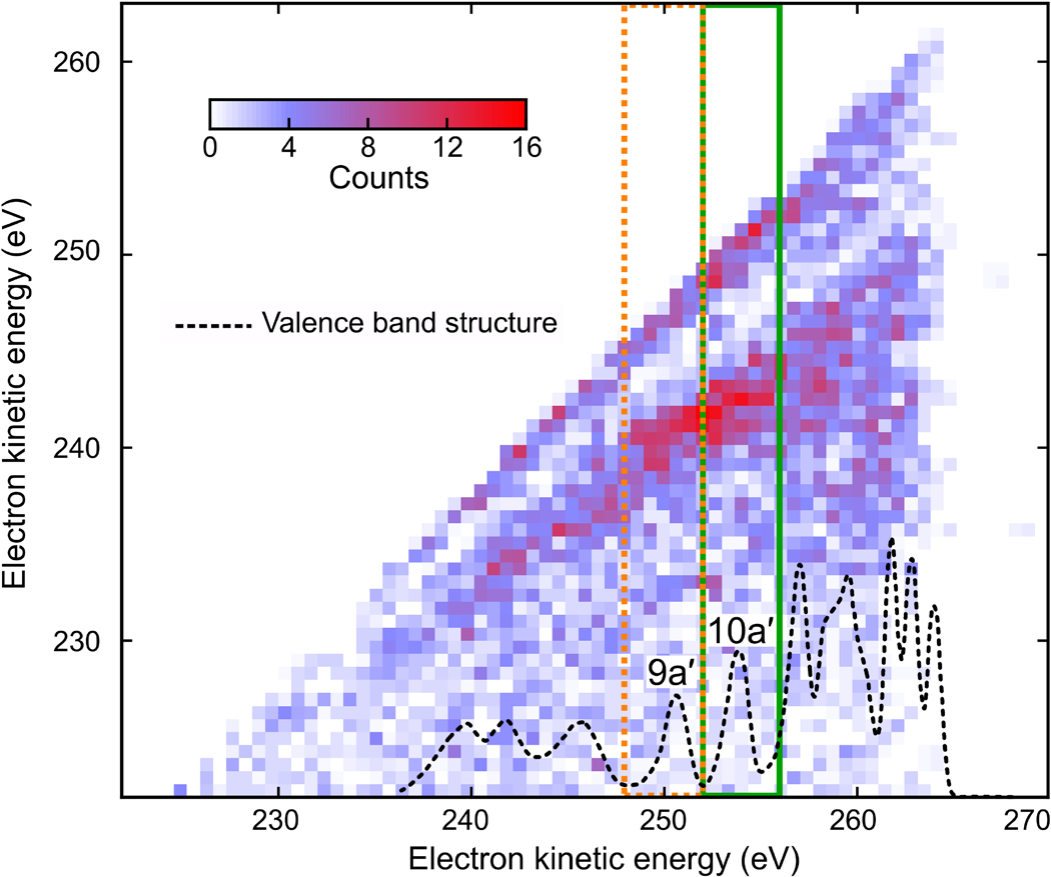
Two-electron coincidence spectroscopy of glycine with 274.0-eV photons. Coincidence map of two-electron detection. Photoelectron (10a′ and 9a′)–Auger electron coincidences following the resonant x-ray transition (1s → 1h) are indicated (green and orange squares). The density of valence states is indicated (dotted black) by using the experimental data reproduced from (*14*). Valence electron emission from x-ray probe-induced sequential double ionization contributes to an uncorrelated background with respect to the (initial) x-ray pump-induced photoionization events, which allows observing the fingerprint of the probe-induced Auger decay. The map also shows an enhanced probability for the detection of two (photo)electrons with an equal kinetic energy, which are the likely result of a false coincidence between two valence ionization events.

We note that, within the time-dependent B-spline ADC(2)x scheme used for simulating the probe step of the experiment, the absolute binding energies of the final dicationic eigenstates are not reproduced with high accuracy and may easily be off by up to ~10 eV [this is in contrast to the accurate cationic eigenenergies obtained using the ADC(2,2) method and used in the simulations of the pump step]. Therefore, we plot the simulated time-dependent electron spectra presented in Fig. 4 (B and C) relative to the theoretically derived phase jump energy (~255 eV), which is above the observed experimental value of (246 ± 2) eV. Furthermore, the ionization dynamics for pump-probe time delays of a few fs, where the x-ray pulses strongly overlap, cannot be accurately described in our model. The reason is that, theoretically, the pump and probe simulations are carried out independently and the probe simulation assumes that the pump-induced ionization is completed. This is also the reason why the onset of the observed electron yield oscillation slightly differs between experiment and theory. We also note that, according to theory, the Auger spectra and the sequential double-ionization spectra do not majorly depend on whether the inner-valence hole is located on the α-carbon or on the carboxyl carbon (see the Supplementary Materials). Although the resonant 4a′ → 9a′ transition energy for inner-shell excitation of the carboxyl group is close to the FEL photon energy and, therefore, considered in the simulation, the FEL spectral bandwidth of less than 1.2 eV (FWHM) is too small to form a coherent superposition of the two C 1s core orbitals 4a′ and 5a′ with high efficiency. Their relative chemical shift is on the order of ~2.9 eV, which gives a time period of ~1.4 fs for the corresponding quantum beat that cannot be resolved in the present experiment. Nevertheless, the contribution from adjacent 9a′ configurations to the observed dynamics is fully taken into account in our simulation and is also visible in static experiments, where two electrons are measured in coincidence (Fig. 5).

Our further simulations take into account the nuclear geometry (but not nuclear dynamics) effects on the temporal evolution of the many-electron wave packet. First, we note that, at an average oven temperature of 160°C in our gas phase experiment, only two conformers commonly referred to as Gly I and Gly III are expected to contribute to the molecular response at a ratio of approximately 2:1 (*12*, *29*). Theoretical investigations have shown that the ionization out of orbital 10a′ gives rise to different many-electron dynamics in Gly III and Gly I, with time scales varying from about 10 to 20 fs, respectively (*23*). Therefore, the time-resolved electron spectroscopic data presented here give the effective oscillation period *T* for the mix of conformers present in the molecular beam. In accordance, we have also calculated the survival probability of the pump-prepared ionic density matrix performing a 2:1 weighted average over the Gly I and Gly III conformers. Furthermore, our simulation of the survival probability also included the effect of the quantum uncertainty in the nuclear geometry because of zero-point energy. To this end, we have used the normal modes of Gly I and Gly III conformers obtained using the Molpro quantum chemistry software package (*30*). Our simulations show that the oscillations of the many-electron wave packet survival probability survive both conformer and the geometry uncertainty averaging for over at least 25 fs, in agreement with the experimental results (see fig. S5). Note that, in some other systems, the effect of the quantum uncertainty in the nuclear positions led to a much faster dephasing of the coherent oscillations of the many-electron wave packets. For example, in isopropanol (*31*), it has been found that while the initial quasi-exponential decay of the inner-valence electron hole is barely affected by the averaging over nuclear geometries, the subsequent quantum revivals of the hole population predicted theoretically at the equilibrium geometry of the neutral molecule are completely destroyed by the averaging over an ensemble of geometries, representing a finite-uncertainty vibrational state. Similarly, in paraxylene (*11*), averaging over the nuclear geometries has been shown to lead to the very efficient dephasing of the hole oscillations because of the coherent population of two electronic states.

## DISCUSSION

This work contributes to the solution of the controversy on the possible time scale of the coherent many-electron dynamics leading to charge migration in ionized polyatomic molecules. Whereas, previously, such dynamics, spanning tens of fs, was only detected indirectly, e.g., via nuclear rather than electronic observables (*3*), here, we present results of two direct modes of detection both based on the electronic observables (see Figs. 2 and 3). We have shown that the electron yield oscillations monitoring the quantum wave packet dynamics of the positive charge in the photoionized glycine cation persist for at least 40 fs. Our work, therefore, puts the idea of the long-time coherent electronic molecular dynamics triggered by photoionization on a much firmer ground than it was previously possible by solely analyzing ionization-induced fragmentation as an indirect signature of charge migration (*3*). Note that another study of charge migration in a completely different (“frustrated Auger”) regime in isopropanol was recently published (*31*) that also uses the spectroscopic technique proposed theoretically in (*12*) to observe highly transient hole states. However, the physical regime that (*31*) addresses is the short-time one, covering only the first few fs of the coherent evolution.

The presented observations of the oscillations in the photo- and Auger electron yields prove the existence of coherent dynamics in photoionized glycine extending over tens of fs. To extract the exact nature of the coherence leading to these observed oscillations in an experiment probing only the electronic degrees of freedom, we require theoretical analysis. Our high-level ADC(2,2) treatment is essential to capture with sufficient accuracy the electronic dynamics, as it provides the proper treatment of the 1h and 2h1p components of the 10a′ state essential for this particularly challenging inner-valence spectral region characterized by partial breakdown of the molecular orbital picture of ionization. A full quantum mechanical simulation of vibronic coherence and vibronic dynamics following glycine photoionization, coupled to this accurate electronic calculation, is presently out of reach. Therefore, we limited the treatment of the nuclear degrees of freedom in our theoretical investigation to averaging over the zero-point energy spread in the nuclear positions, which has been previously shown to lead to dephasing of the electronic coherence (*11*). Our ab initio theoretical investigation showed that the period of the observed initial oscillations (~20 fs) is fully consistent with the Born-Oppenheimer picture of an electronic process taking place over a distribution of nuclear geometries.

While the oscillation period observed in the electronic signals is also similar to that of the glycine C═O stretch, it cannot be explained by a coherent excitation of vibrations in the cation on a potential energy surface of a single electronic state, because coherent population of several bands of electronic states has been firmly established by the pump simulation (see fig. S1). At the same time, it would be naive to attribute the observed coherence to a purely electronic one, as modeled here within the Born-Oppenheimer picture. Energy spacings in the energy region of partial breakdown of the molecular orbital picture to which the 10a′ states belong are of the same order of magnitude as some of the vibrational quanta, and the two degrees of freedom are expected to strongly couple, resulting in quantum eigenstates represented by linear superpositions of electronic states dressed by vibrational excitations. Nonadiabatic dynamics and its effect on the vibronic coherence have been studied by Mukamel and co-workers in the case of core ionization or excitation (*32*–*35*), where breakdown of the molecular orbital picture is not typical. In our work, we experimentally probe, via electronic degrees of freedom, the cationic state coherence resulting from the inner-valence ionization. Our many-electron simulations show that this coherent oscillation period of the probe electron signal can be successfully approximated by the coherent dynamics of the Born-Oppenheimer electronic states averaged over the zero-point energy distribution of the nuclear geometries. However, the full coherence brought to life by the few-fs ionization of glycine in the inner-valence region is, in all probability, of mixed electronic and vibrational character. Full characterization of this coherence theoretically within the highly computationally demanding inner-valence energy region is currently beyond reach and should be the subject of future theoretical studies. One can, nevertheless, speculate on the basis of the experimental results presented here that the long time span of the coherent signal oscillations observed in this work may well be a result of the similarity in the time scales of the electronic and vibrational dynamics.

## MATERIALS AND METHODS

### Experimental design

**Fig. 6.**
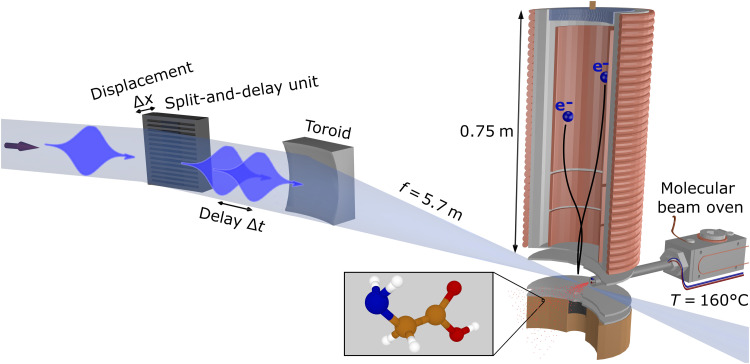
Schematic setup. Experimental setup for the single-color, fs x-ray pump-probe measurements of glycine at FLASH2.

The experiment was carried out at the FL24 beamline of the FLASH2 FEL at Deutsches Elektronen-Synchrotron DESY in Hamburg (*18*). The FEL is operated in 10-Hz bursts with trains of 400 electron bunches each with 1-μs separation, in a special short-pulse mode with a low electron bunch charge of a few tens of picocoulombs. Each bunch generates a coherent FEL pulse with sub–5-fs duration at 274.0-eV (off-resonance) and 272.7-eV (on-resonance) photon energy and an average pulse energy of about 5 μJ (4000 photon pulses per second in total). The FEL wavelength is tuned by changing the gap between the magnetic poles of the FEL undulator magnets, while the electron beam energy is kept constant at 1.24 GeV. The scheme of the experimental setup is shown in Fig. 6.

We used a split-and-delay unit consisting of two interleaved lamellar mirrors in the unfocused beam to generate two replicas of collinearly propagating FEL pulses with a controllable delay (*17*). The resulting pump-probe sequences were focused into the interaction region by a toroidal mirror with a focal length of about 5.7 m. Both the lamellar-mirror pair and the focusing mirror were nickel-coated and operated at 8° angle of incidence to the surface.

For the experiments, >98.5% glycine was acquired from Sigma-Aldrich and used without further purification. The glycine molecules were delivered to the interaction region by evaporation of glycine powder at 160°C in a resistively heated oven. The resulting vapor pressure of glycine in the 10^−3^-mbar range produced an effusive beam of sufficient density.

The photoelectrons and Auger electrons generated in the interaction region were detected by a microchannel plate (MCP) detector in a magnetic bottle–type TOF electron spectrometer. It allows the application of retardation fields to provide the necessary resolution for fast electrons with several hundred electron volt kinetic energy. The MCP signal was digitized by a fast analog-to-digital converter and stored in the FLASH data acquisition system. The electron kinetic energies were calibrated with the well-known photoelectron spectrum of argon. The setup also allowed for simultaneous measurement of TOF mass spectra, which were calibrated by means of mass spectrometry using different rare gases.

To generate the shortest possible pulses, FLASH uses a separate photoinjector laser optimized for low bunch-charge operation. The low charge enables a very high bunch compression because of smaller space-charge effects. The short electron bunch reduces the number of individual longitudinal modes present in the FEL pulses, in the ideal case, down to a single mode. This is referred to as single-spike self-amplified spontaneous emission (SASE) or single-mode SASE operation. It increases the longitudinal coherence as the slippage between the electrons and the light in the undulator is larger than the bunch length so the light emitted from the tail of the electron bunch reaches the head of the bunch within a few gain lengths and clearly before saturation. Single-spike operation delivers sub–5-fs pulses with a high degree of longitudinal coherence from a SASE source as the whole electron bunch interacts with the light from the same mode (*20*, *21*).

**Fig. 7.**
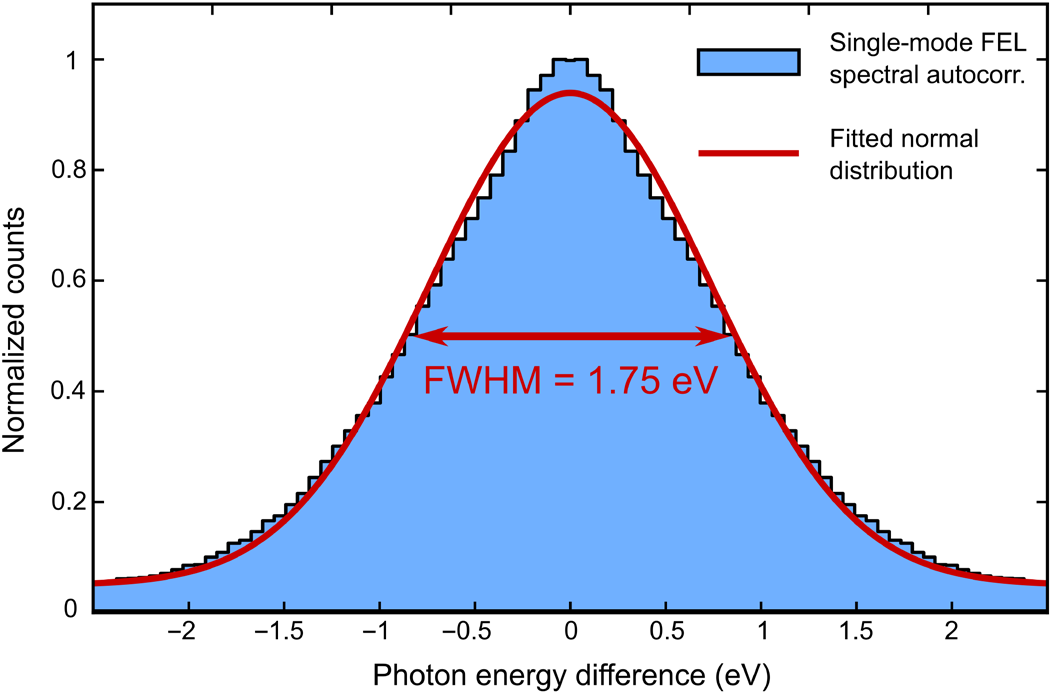
FEL photon energy autocorrelation for the dataset shown in Fig. 3. Photoionization from the argon 2p_1/2_ and 2p_3/2_ orbitals was used to infer the FEL photon energy from four TOF spectrometers.

During our beamtime, we used the Online Photoionization Spectrometer (OPIS) system in the FLASH2 tunnel for photon diagnostics (*18*). OPIS consists of four electron TOF spectrometers arranged in a cross shape to monitor the FEL wavelength and bandwidth by detecting photoelectrons from a thin rare gas target. To evaluate the FEL spectral bandwidth, we correlated events in which two electrons were detected in opposite-lying TOF spectrometers. Each photoelectron was assigned to either the argon 2p_1/2_ or 2p_3/2_ orbitals, and only matching pairs originating from the same orbital of two atoms within one FEL shot were considered. For the dataset shown in Fig. 3, it results in a total number of ~2.6 × 10^7^ electron pairs, which are plotted in Fig. 7. For these pairs, the differences of their respective photoelectron kinetic energies were calculated and are shown as a histogram. The width of the equidistant energy bins was chosen to approximate the resolution of the analog-to-digital converter with a sampling rate of 7 GHz. This procedure results in an autocorrelation of the FEL shots in the spectral domain. Assuming a Gaussian distribution, the FEL spectral bandwidth during the data acquisition in the glycine experiment can be calculated to be 1.24 eV (FWHM) or about 0.45% of the central photon energy tuned in the range between 269 and 281 eV. We note that this number still includes the instrument functions of the OPIS spectrometers as well as a broadening by the finite-size beam profile and pointing jitter of the FEL and is therefore an upper limit.

### Data analysis and fitting

In Fig. 1A, each spectrum was measured for about 5 to 6 min and normalized by the respective number of detected electrons to account for any fluctuations in FEL pulse energy and other FEL parameters. The average FEL wavelength per bunch train drifted less than 0.1% around the central wavelengths. Instead of using the Jacobi transformation for the electron TOF–to–kinetic energy conversion, the equidistant TOF bins were resampled to equidistant energy bins with a linear filter to avoid aliasing effects. The energy bin size of 0.3 eV was chosen to match the energy width of the first TOF bin in the spectra shown in Fig. 1A.

For the false color plot in Fig. 1A, the spectra were additionally smoothed by applying a moving average over a window of 2.5-eV electron kinetic energy to suppress noise and emphasize the general trends. Between the spectra recorded at different photon energies, a linear interpolation was applied.

In Fig. 3A, the electron spectra were divided by the total number of detected electrons for each delay to account for any FEL fluctuations, and, afterwards, the residual gas contribution was subtracted. Here, the measurement time was 10 min per delay. The *y* axis shows the relative change of the electron yield compared to the average yield in percent. All four oscillations were fitted with a sinusoidal curve of the form

$$f_{i}(∆t)=a_{i}\cdot \text{sin}(\omega_{i}\cdot ∆t+\varphi_{i})$$ (3)

where *a_i_* is the amplitude, φ*_i_* is a phase offset, $\omega_{i}=\frac{2\;\pi }{T_{i}}$, and *T_i_* is the period of the oscillation for *i* ∈ [1, 4]. As the average spectrum over all delays was subtracted for every single delay, the resulting relative change of the electron yield in percent naturally oscillates around 0. The oscillation amplitudes, periods, phases, and the goodness $R_{i}^{2}$ of the fits are summarized in Table 1.

**Table 1.** Fitting parameters of sinusoidal curves of the form of **Eq. 3.** The oscillation amplitudes, periods, phases, and the goodness $R_{i}^{2}$ of the fits describing the experimental data shown in Fig. 3A are summarized.

**Energy** **range**	**Amplitude**	**Period**	**Phase**	**Goodness**
259 to 263 eV	*a*_1_ ≈ (28 ± 11)%	$T_{1}\approx (20.6_{-2.8}^{+3.8})$fs	φ_1_ ≈ (0.0 ± 0.2)π	$R_{1}^{2}\approx 0.58$
252 to 256 eV	*a*_2_ ≈ (26 ± 14)%	$T_{2}\approx (18.8_{-3.4}^{+5.4})$ fs	φ_2_ ≈ ( − 0.1 ± 0.3)π	$R_{2}^{2}\approx 0.37$
235 to 239 eV	*a*_3_ ≈ (16 ± 6)%	$T_{3}\approx (21.4_{-3.9}^{+6.2})$ fs	φ_3_ ≈ (1.2 ± 0.3)π	$R_{3}^{2}\approx 0.55$
231 to 235 eV	*a*_4_ ≈ (24 ± 9)%	$T_{4}\approx (20.5_{-3.2}^{+4.6})$ fs	φ_4_ ≈ (1.1 ± 0.3)π	$R_{4}^{2}\approx 0.58$

For Fig. 3C, the kinetic energy range of 224 to 264 eV was split into overlapping intervals, with 4-eV width in steps of 1 eV. Each of these delay-dependent electron spectra was fitted according to the formula used for Fig. 3A. The result of the 37 individual fits is plotted in Fig. 3C, with the central energy of the 4-eV energy range on the *y* axis.

All of the kinetic energy ranges from Fig. 3C were also fitted with sinusoidal curves of the same period but free phase and amplitudes according to

$$f_{i}(∆t)=a_{i}\cdot \text{sin}(\omega \cdot ∆t+\varphi_{i})$$ (4)

where *a_i_* is the amplitude and φ*_i_* is a phase offset for *i* ∈ [1, 37], $\omega =\frac{2\;\pi }{T}$, and *T* is the period of the oscillation. The resulting period *T* of this fit is indicated in Fig. 3 (A and E) as black dashed lines. The shown error values are the mean of the upper and lower 95% for the 37 individual sinusoidal fits of overlapping energy intervals.

The fitted period presented in Fig. 3 within two SDs, $T=(19.6_{-1.4}^{+2.2})$ fs, corresponds to an energy level separation of the two cationic eigenstates Ψ*_i_* and Ψ*_j_* of ∆*E* ≈ (0.21 ± 0.02) eV according to Eq. 2.

To identify the somewhat weaker probe-induced Auger channel at the central photon energy of 274.0 eV (off-resonance) experimentally demonstrating the element specificity and orbital selectivity of the 1s → 1h resonant transition, we selected FEL shots in which two electrons were detected in coincidence. Of ~6 × 10^5^ electrons created in the interaction of glycine molecules with ~1 × 10^8^ FEL pulse pairs, we identified ~7600 coincidences integrated over all time delays. In Fig. 5, the kinetic energy of the first (faster) and second (slower) electron is plotted on the *x* and *y* axis, respectively. Among the many possible multielectron excitation pathways and decay channels creating doubly charged final states, particularly those cascades starting with photoionization of the 9a′ and 10a′, molecular orbitals are significantly enhanced in the coincidence map as indicated in Fig. 5. The signature of a pump-probe event including Auger decay is the observation of a photoelectron at kinetic energy corresponding to valence ionization of 10a′ (or 9a′) in combination with an Auger electron that is downshifted in energy by the difference of the first and second ionization potential.

The coincidence event map is obtained as follows: Every single-shot kinetic energy spectrum with two electrons is represented by a row vector *S*(*E*_1_, …, *E_N_*), where 1…*N* are the indices of energy bins. It is transformed into an *N* × *N* matrix *M* = *S^T^S*. Then, all these single-shot matrices, which are symmetric with respect to the diagonal, are summed up, and the diagonal line, together with the upper triangle, is removed for clarity. Columns and rows of the resulting matrix represent the energies of the first electron and the second electron, respectively. The values of elements *p_mn_* of the matrix contain the number of double events, where the first electron had the kinetic energy *E_n_* and the second electron had the kinetic energy *E_m_*.

On the basis of the knowledge gained from the coincidence map of the static experiment discussed above, we searched our data recorded at the FEL photon energy of 272.7 eV (on-resonance) for two or more electron detection simultaneously to the appearance of the doubly charged glycine parent ion (Gly^2+^) per FEL shot. Then, we filtered these triple events, fixing the kinetic energy of one electron to the valence ionization of 10a′ within a detection window of ±5 eV. The resulting electron yield is divided by the total number of detected electrons for each delay to account for any FEL fluctuations. Afterwards, the difference between this signal and the average over the full delay scan is plotted to derive the relative changes of the electron yield as a function of x-ray pump x-ray probe delay in 1-fs steps, which is shown in Fig. 2. Continuous wavelet transform (CWT) allows us to study the magnitude evolution of a nonstationary signal at scaling frequencies. We used generalized Morse wavelets as described by Lilly and Olhede (*36*) with symmetry parameter γ = 3 and time-bandwidth product *P*^2^ = 60. The CWT uses 48 voices per octave. The “cone of influence” (COI), where part of the wavelet in time domain extends past the finite recorded experimental signal trace, is given as a darker shaded area. In the COI area, artificial edge effects disturb the frequency analysis and, therefore, peak structures in the shaded area of the color plot are likely underestimated. The boundaries of the COI are chosen as the points, where the autocorrelation magnitude of the respective wavelet decays by 1/*e*.
