# Supplementary material for: Electronic quantum coherence in glycine molecules probed with ultrashort x-ray pulses in real time
Source: Sci Adv. 2022 Jun 1;8(22):eabn6848. doi: 10.1126/sciadv.abn6848 (PMC9159702; doi:10.1126/sciadv.abn6848)
Supplement: Supplementary file 1 — Supplementary Text Figs. S1 to S5 [file sciadv.abn6848_sm.pdf]

Science Advances
AAAS

Supplementary Materials for

Electronic quantum coherence in glycine molecules probed with ultrashort x-ray pulses in real time

David Schwickert *et al.*

Corresponding author: Tim Laarmann, tim.laarmann@desy.de

Sci. Adv. **8**, eabn6848 (2022)
DOI: 10.1126/sciadv.abn6848

The PDF file includes:

Supplementary Text
Figs. S1 to S5
Legend for movie S1

Other Supplementary Material for this manuscript includes the following:

Movie S1

Supplementary Text

S1. Theoretical methods.

Advanced theoretical modeling and simulation of the photoionization dynamics of the glycine molecule triggered by the x-ray pump and probe laser pulses is performed here, from first-principles, within the framework of the cutting-edge time-dependent B-spline restricted correlation space (RCS) – algebraic diagrammatic construction (ADC) *ab initio* methods (24-26). Both interactions of the neutral glycine molecule and its cation with the pump and probe x-ray femtosecond pulses, respectively, are described from first-principles. The simulations of photoionization during the pump and probe steps are carried out, within the frozen-nuclei approximation, at the equilibrium geometry of the Gly I conformer. We also analyze the effect of the geometry-spread of the ground state nuclear wavefunction on the survival of the quantum electronic coherences in the 10a' and 9a' ionized bands (see Section S3).

S1.1. First ionization of the neutral species by the x-ray pump pulse.

Within B-spline RCS-ADC, the single-electron orbitals are expanded in a multicenter B-spline basis set (24, 28), designed to accurately describe both bound and ionized states of the neutral molecule. The resulting Hilbert space of Hartree-Fock (HF) virtual orbitals (ϕ_a), is subsequently partitioned into two orthonormal subspaces: the restricted correlation space (RCS) (χ_α), designed to accurately describe the localized short-range correlation of the system, and its orthonormal complement, the ionization space (IS) (ψ_μ), which describes the long-range part of the photoelectron wavefunction and complements the RCS in order to represent the electronic wavefunction over the entire spatial region (24). Here and in what follows, the $\alpha, \beta, \ldots$ and $\mu, \nu, \ldots$ indices refer to the virtual space (unoccupied) RCS and IS orbitals, respectively, whereas i, j, k, … , indicate the occupied HF molecular orbitals.

Within the time-dependent (TD) B-spline RCS-ADC(n) approach to molecular photoionization dynamics (25, 26), the 3D time-dependent Schrödinger equation (TDSE) for an N-electron polyatomic molecule interacting with an ultrashort laser pulse

$$i\hbar \frac{\partial \mid \Psi^N(t) \rangle}{\partial t} = \hat{H}^N_{Pump}(t) \mid \Psi^N(t) \rangle \qquad (1)$$

is solved by expanding the TD many-electron wavefunction in the basis of the ground and excited RCS-ADC(n) states (25, 26)

$$\mid \Psi^N(t) \rangle = \sum_{m,\mu} c_{m,\mu}(t)\ \hat{a}^\dagger_\mu \mid \Phi_m^{N-1,RCS}\rangle + \sum_{I_{RCS}} c_{I_{RCS}}(t) \mid \tilde{\Psi}^N_{I_{RCS}}\rangle^{[n]} + c_0(t) \mid \Psi_0^{RCS}\rangle^{[n]}. \qquad (2)$$

Here $\mid \Psi_0^{RCS}\rangle^{[n]}$ is the n-th order RCS correlated ground state, and $\mid \tilde{\Psi}^N_{I_{RCS}}\rangle^{[n]}$ indicates the excited intermediate states of the nth-order ADC(n) scheme, built using the single-particle RCS basis. The ansatz of Eq. (2) includes a full description of electron correlation effects, such as shake-up processes, breakdown of the molecular orbital (MO) picture and inter-channel couplings in the continuum, which can play an essential role both during the ionization event and the post-ionization charge dynamics.

Within the extended second-order ADC(2)x scheme for many-electron excitation used in this work, the excited (N)-electron intermediate states span the configuration space consisting of one-hole – one-particle (1h1p) $\left(|\,\tilde{\Psi}_{ai}^{N}\rangle^{[n]}\right)$ and two-hole – two-particle (2h2p) $\left(|\,\tilde{\Psi}_{\alpha\beta ij}^{N}\rangle^{[n]}\right)$ excitation classes built on top of the RCS correlated ground state (24). The first term on the right-hand side of Eq. (2) describes the IS configuration states of B-spline RCS-ADC, which take the form of ionization-channel-specific product-states and reads $|\,\Psi_{\mu,m}^{N}\rangle = \hat{a}_{\mu}^{\dagger}\,|\,\Phi_{m}^{N-1,RCS}\rangle$, where $\hat{a}_{\mu}^{\dagger}$ is the creation operator of an electron in the IS molecular orbital $\psi_{\mu}(r)$, and $|\,\Phi_{m}^{N-1,RCS}\rangle$ denotes the RCS-ADC ionic eigenstates. In this work, the latter have been calculated using the ADC(2,2) (27) method for (N-1)-electron systems.

Thus, the $|\,\Phi_{m}^{N-1,RCS}\rangle$ are obtained by diagonalizing the ionic Hamiltonian calculated at the ADC(2,2) level of theory and using the single-particle RCS basis set

$$\hat{H}_{ADC(2,2)}^{N-1,RCS}\,|\,\Phi_{m}^{N-1,RCS}\rangle = I_{m}^{p}\,|\,\Phi_{m}^{N-1,RCS}\rangle, \quad (3)$$

where the ionization energy is given by $I_{m}^{p} = E_{m}^{N-1} - E_{0}^{RCS}$ and E_{0}^{RCS} is the energy of the RCS ground state. Within the RCS-ADC(2,2) scheme, the contributions to electron correlation of one-hole (1h) and two-hole – one-particle (2h1p) configurations, is taken into account in a better-balanced way compared to both ADC(2)x and ADC(3) methods (27), making the approach more appropriate for spectral region with strong MO picture breakdown. The ionic states are expanded into 1h, 2h1p and 3h2p configurations derived from the correlated RCS ground state of the neutral molecule

$$|\,\Phi_{m}^{N-1,RCS}\rangle = \sum_{i} V_{i,m}^{+}\,|\,\tilde{\Phi}_{i}^{N-1}\rangle + \sum_{\alpha ij} V_{\alpha ij,m}^{+}\,|\,\tilde{\Phi}_{\alpha ij}^{N-1}\rangle + \sum_{\alpha\beta ijk} V_{\alpha ij,m}^{+}\,|\,\tilde{\Phi}_{\alpha\beta ijk}^{N-1}\rangle. \quad (4)$$

The total time-dependent Hamiltonian of Eq. (1) reads

$$\hat{H}_{Pump}^{N}(t) = \hat{H}_{RCS-ADC}^{N} + \hat{D}_{RCS-ADC}^{N} E_{Pump}(t) - \mathrm{i}\hat{W}, \quad (5)$$

where a complex absorbing potential (CAP) of the form $\hat{W} = \eta(r - r_{CAP})^{2}$ $(r \geq r_{CAP})$ is used to eliminate wave packet reflections from the boundaries of the B-spline radial grid. The laser-molecule interaction driven by the x-ray pump electric field $\left(E_{Pump}(t)\right)$ is described within the dipole approximation in the length form, and $\hat{H}_{RCS-ADC}^{N}$ and $\hat{D}_{RCS-ADC}^{N}$ are the representation of the shifted field-free Hamiltonian $\hat{\hat{H}} = \hat{H} - E_{0}^{RCS}$ and the dipole operator $\hat{D}$, respectively, in the space of RCS-ADC intermediate states (see Eq. (2)). These matrices contain as sub-blocks the conventional ADC matrices computed within the RCS-based excitation space, and are further augmented with the blocks corresponding to the product-states, which describe ionization and interaction in the continuum (24-26). All the blocks of the $\hat{H}_{RCS-ADC}^{N}$ matrix are evaluated at the ADC(2)x level of theory, with the only exception of the Hamiltonian terms describing the energy of the (N-1)-electron ionic states, evaluated at the ADC(2,2) level of theory.

The time propagation of the unknown coefficients of the neutral many-electron wavefunction Eq. (2) is performed by means of the Arnoldi/Lanczos algorithm (25, 26). During the simulation of the pump step, we have included into the expansion of the many-electron wavefunction only the open ionization channels with energy up to the double ionization threshold (DIP) and characterized by an (ionization) spectral intensity value (see ref. (24, 25)) greater than 1%. These states will be denoted in the following as $|\,\Phi_{n,\mathrm{Pump-ionized}}^{N-1,RCS}\rangle$. Doing so guarantees that all the ionic states that can be effectively populated in the x-ray pump ionization, and consequently play the

main role in the ensuing many-electron dynamics taking place in the molecular cation, are accounted for (25, 26). The contribution of ionic states with a smaller spectral-intensity value, as well as of deeper core-ionization channels, was indeed found to be negligible.

The pump-triggered coherent electron dynamics is obtained by calculating, in the basis of ionic eigenstates $| \Phi^{N-1,RCS}_{m,\ Pump-ionised}\rangle$, the time-dependent reduced ionic density matrix (R-IDM) $\hat{\rho}^{R-IDM}(t)$ of the molecular ion emerging from the femtosecond pump-ionization step. This is achieved by tracing out the unobserved photoelectron degree of freedom from the total time-dependent density matrix of the N-electron system

$$\hat{\rho}^{R-IDM}(t) = \mathrm{Tr}_{\mu}\,[|\,\Psi^N(t)\,\rangle\langle\,\Psi^N(t)\,|]. \tag{6}$$

Doing so, and using the many-electron states of Eq. (2) within the TD RCS-ADC(n) framework, yields in the basis of ADC(2,2) ionic eigenstates

$$\hat{\rho}^{R-IDM}_{m,n}(t) = \sum_{\mu} c_{m\mu}(t)\left[c_{n\mu}(t)\right]^* + 2e^{i(I_n^p - I_m^p)t}\int_{-\infty}^{t}\sum_{\mu,\nu} w_{\nu,\mu}c_{m\mu}(t')[c_{n\nu}(t')]^* e^{-i(I_n^p - I_m^p)t'}dt', \tag{7}$$

where the latter term corrects for the loss of norm introduced by the CAP (24), I_m^p is the ionization potential of the ionic state m and $w_{\nu,\mu}$ is the CAP matrix element between photoelectron IS orbitals μ and ν. From now on we shall omit the R-IDM superscript and denote the reduced ionic density matrix as $\rho_{m,n}$.

The R-IDM fully describes the mixed ionic state prepared by attosecond molecular ionization: the TD population of the different ionic eigenstates is given by the diagonal elements

$$p_n(t) = |\rho_{n,n}(t)|, \tag{8}$$

while the off-diagonal ones $\rho_{m,n}$ are related to the degrees of quantum electronic coherence, $0 \leq G_{m,n} \leq 1$, between any pair of populated ionic channels m and n

$$G_{m,n}(t) = \frac{|\rho_{m,n}(t)|}{\sqrt{p_m(t) * p_n(t)}}. \tag{9}$$

In addition, the relative phases $\varphi_{m,n}$ between the partially-coherently populated eigenstates of energies I_m^p and I_n^p are extracted from the phases of the R-IDM off-diagonal matrix elements and read

$$\varphi_{m,n} = \arg\left[\rho_{m,n}(t)\, e^{+i(I_m^p - I_n^p)t}\right]. \tag{10}$$

The relative phase matrix is antisymmetric, i.e. $\varphi_{m,n} = -\varphi_{n,m}$, as follows from the hermiticity of the density matrix ($\rho_{n,m} = \rho^*_{m,n}$).

Finally, diagonalization of the ionic density matrix yields the so-called Schmidt decomposition (26). The latter represents a powerful tool to analyze the produced mixed state of the cationic system, allowing one to express it as a fully-incoherent sum of several (N_{ionic}) fully quantum-coherent pure states, each of them populated with weight r_m and represented by the projection operator $\hat{P}_m$:

$$\hat{\rho}(t) = \sum_{m=1,\ N_{ionic}} r_m(t)\,\hat{P}_m(t) = \sum_{m=1,\ N_{ionic}} r_m(t)\ |\ \Phi_m^{\rho}(t)\rangle\langle \Phi_m^{\rho}(t)\ | \tag{11}$$

This operation is called purification of the density matrix.

S1.2. Second ionization of the cationic species by the x-ray probe pulse.

In this work, we explicitly simulate the interaction of the pump-ionized system with the probe pulse. Doing so allows us to calculate the time-delay dependence of probe-induced electron emission from the pump-ionized glycine cation, thus obtaining a realistic description of the ultrafast electronic observables measured in the experiment.

The formal validity of the presented theoretical framework relies on non-overlapping pump and probe pulses. The reason for this restriction is that, while the current B-spline RCS-ADC implementation can accurately treat many-electron dynamics with one photoelectron in the continuum, it cannot yet afford the modeling of two photoelectrons in the continuum at the same time. The latter, which would in fact be more appropriate in the case of overlapping pump and probe pulses, would also require one to extend the expansion of the many-electron RCS-ADC wavefunction (Eq. (2)) by states of the type $\hat{a}_{\mu}^{\dagger}\hat{a}_{\nu}^{\dagger}\ |\ \Omega_m^{N-2,RCS}\rangle$, thus posing an extra demand on the computation by means of the TD RCS-ADC machinery. However, in practise, this only limits the range pump-probe scenarios that can be numerically tackled to the ones corresponding to time-delays greater than the duration of the x-ray pulses involved $\tau_d > T_{Pump}/2,$ i.e. in this case corresponding to delay times of 1.3 fs or longer.

The time-dependent description of the cation-probe interaction is performed by solving the time-dependent von Neumann equations (26) for the characterized reduced ionic density matrix (Eq. (7)) interacting with the probe laser field

$$\frac{d}{dt}\,\hat{\rho}(t) = -\frac{i}{\hbar}\left[\hat{H}_{Probe}^{N-1}(t)\,,\hat{\rho}(t)\right]. \tag{12}$$

Eq. (12) is solved by using the B-spline RCS-ADC method, which we extended here to describe the photoionization dynamics of (N-1)-electron systems. The solution provides an explicit description of the ionization continua of the dication as well as of the Auger decay process, and both photoemission channels are included in the calculation. In particular, this equation can be solved by taking advantage of the Schmidt decomposition Eq. (11) of the ionic density matrix upon the pump-ionization step. It allows us to tackle the solution of Eq. (12) by propagating $N_{ionic}^{t=0}$ independent TDSE,

$$i\,\hbar\ \frac{\partial\ |\ \Phi_m^{\rho}(t)\rangle}{\partial t} = \hat{H}_{Probe}^{N-1}(t)\ \ |\ \Phi_m^{\rho}(t)\rangle \qquad m = 1,\ldots\ldots,N_{ionic}^{t=0} \tag{13}$$

corresponding to the individual pure ionic states obtained by the purification of the initial (before the interaction with the probe pulse) ionic density matrix prepared by the pump pulse. The time-dependent $|\ \Phi_m^{\rho}(t)\rangle$ states can be expanded in the basis of (N-1)-electron RCS-ADC states as

$$|\ \Phi_m^{\rho,N-1}(t)\rangle = \sum\nolimits_{m,\mu} c_{m,\mu}(t)\ \hat{a}_{\mu}^{\dagger}\ |\ \Omega_m^{N-2,RCS}\rangle + \sum\nolimits_{I_{RCS}} c_{I_{RCS}}(t)\ |\ \tilde{\Phi}_{I_{RCS}}^{N-1}\rangle^{[n]}. \tag{14}$$

This expression can be further simplified, and renormalized in terms of cationic eigenstates, by expressing the $|\ \tilde{\Phi}_{I_{RCS}}^{N-1}\rangle$ RCS configurations in terms of the RCS ionic eigenstates

$| \Phi^{N-1,RCS}_{n',Pump-ionized} \rangle$ and $| \Phi^{N-1,RCS}_{n'',Core} \rangle$. One derives

$$| \Phi^{\rho,N-1}_{m} (t) \rangle = \sum_{n,\mu} c_{m;n\mu}(t)\, \hat{a}^{\dagger}_{\mu} \,|\, \Omega^{N-2,RCS}_{n} \rangle + \\ + \sum_{n'} c_{m;n'}(t) \,|\, \Phi^{N-1,RCS}_{n',\text{Pump-ionized}} \rangle + \sum_{n''} c_{m;n''}(t) \,|\, \Phi^{N-1,RCS}_{n'',Core} \rangle. \quad (15)$$

In Eq. (15) we have thus expressed the wavefunction of the time-dependent pure-state channel $| \Phi^{\rho,N-1}_{m} (t) \rangle$ as an expansion over the full electronic spectrum of the ionic subsystem, including both its low-energy bound excitations, i.e. the valence-ionized states populated by the pump pulse, the C(1s) core-ionized states, which were not populated in the pump-step simulation, and the electronic continua of the valence-doubly-ionized states.

The time-dependent ionic Hamiltonian $\hat{H}^{N-1}_{Probe}(t)$ in the dipole approximation is given by

$$\hat{H}^{N-1}_{Probe}(t) = \hat{H}^{N-1}_{RCS-ADC} + \hat{D}^{N-1}_{RCS-ADC} E_{Probe}(t) - \mathrm{i}\widehat{W}, \quad (16)$$

where $\hat{H}^{N-1}_{RCS-ADC}$ and $\hat{D}^{N-1}_{RCS-ADC}$ are again the representations of the shifted field-free ionic Hamiltonian and the dipole operator, respectively, in the (N-1)-electron configuration space spanned by all the ionic states of Eq. (15). In this work, the RCS core-ionized states are calculated at the ADC(2)x level of theory employing the core-valence approximation, i.e.

$\langle \Phi^{N-1,RCS}_{\text{n}',\text{Pump-ionized}} \,|\, \hat{H}^{N-1}_{RCS-ADC} \,|\, \Phi^{N-1,RCS}_{n'',Core} \rangle = 0$, and read

$$\hat{H}^{N-1,RCS}_{ADC(2)x} \,|\, \Phi^{N-1,RCS}_{n'',Core} \rangle = I^{p}_{n''} \,|\, \Phi^{N-1,RCS}_{n'',Core} \rangle \;;\; I^{p}_{n''} = E^{N-1}_{n''} - E^{RCS}_{0} \quad (17)$$

$$| \Phi^{N-1,RCS}_{n'',Core} \rangle = \sum_{i} V^{+}_{i,n''} \,|\, \tilde{\Phi}^{N-1}_{i} \rangle + \sum_{\alpha ij} V^{+}_{\alpha ij,n''} \,|\, \tilde{\Phi}^{N-1}_{\alpha ij} \rangle. \quad (18)$$

In Eq. (18) each of the ADC configurations in the expansion features at least one hole-index (i, j) correspond to either the 4a' or 5a' C(1s) occupied molecular orbital; Eq. (18) thus describes the core-ionized states of the glycine cation in the energy range of the carbon K-edge.

The first term on the right-hand side of Eq. (15) describes the IS configuration states of B-spline RCS-ADC for (N-1)-electron systems. These states take the form of ionization-channel-specific product-states

$$| \Phi^{N-1}_{\mu,n} \rangle = \hat{a}^{\dagger}_{\mu} \,|\, \Omega^{N-2,RCS}_{n} \rangle \quad (19)$$

built upon the RCS-ADC eigenstates of the dication $| \Omega^{N-2,RCS}_{n} \rangle$. The latter are obtained by diagonalizing the dicationic ADC Hamiltonian $\hat{H}^{N-2,RCS}_{ADC(1)}$ computed using the single-particle RCS basis set

$$\hat{H}^{N-2,RCS}_{ADC(1)} \,|\, \Omega^{N-2,RCS}_{n} \rangle = DIP_{n} \,|\, \Omega^{N-2,RCS}_{n} \rangle, \quad (20)$$

where the double-ionization energy is given by $DIP_{n} = E^{N-2}_{n} - E^{RCS}_{0}$. In this work, the eigenstates of the dication (produced by the probe pulse) $| \Omega^{N-2,RCS}_{n,\text{Probe-ionized}} \rangle = \sum_{ij} V^{2+}_{ij,n} \,|\, \tilde{\Omega}^{N-2}_{ij} \rangle$ are described within the ADC(1) scheme for (N-2)-electron systems, and are expanded into 2h $| \tilde{\Omega}^{N-2}_{ij} \rangle$ configurations derived from the correlated ground state of the neutral molecule. They are thus described up to first order in the many-body perturbation theory.

The remaining blocks $\langle \Phi^{N-1,RCS}_{n',\text{Pump-ionized}} \,|\, \hat{\hat{H}}^{N-1}_{RCS-ADC} \,|\, \Phi^{N-1}_{\mu,n} \rangle$,

$\langle\, \Phi^{N-1,RCS}_{n',Core} \mid \hat{\hat{H}}^{N-1}_{RCS-ADC} \mid \Phi^{N-1}_{\mu,n} \rangle$ and $\langle\, \Phi^{N-1}_{\nu,n'} \mid \hat{\hat{H}}^{N-1}_{RCS-ADC} \mid \Phi^{N-1}_{\mu,n} \rangle$ are calculated at the ADC(2)x level of theory.

The initial condition for the solution of Eq. (13) reads

$$\mid \Phi^{\rho,N-1}_{m}\,(t=\tau_d)\,\rangle = \sum\nolimits_{n'} c_{m;n'}(t=0)\, e^{-\frac{i}{\hbar} I^{p}_{n'}\,\tau_d} \mid \Phi^{N-1,RCS}_{n',\text{Pump-ionized}} \rangle\,. \qquad (21)$$

It is important to note that the validity of the presented modeling of the cation-probe interaction requires the effect of the interaction with the x-ray probe pulse, on the ensuing many-electron dynamics of interest, to dominate over the residual interaction of the produced cationic system with the emitted photoelectron. For the aforementioned values of the pump-probe time-delay used here, this is indeed the case, as confirmed by the fact that the ionic density matrix $\hat{\rho}(t)$ stabilizes within the first 300 as after the pump ionization event. This is a result of the high kinetic energy of the photoelectron leaving the molecular region, and justifies the neglect of the pump-emitted photoelectron during the description of the probe step, i.e. at times greater than 1.3 fs.

Analogously to the pump-step procedure, we calculate here the time-dependent reduced dicationic density matrix (R-DIDM) $\hat{\rho}^{R-DIDM}(t)$ emerging from the probe-ionization step, by tracing out the unobserved photoelectron degree of freedom from the total time-dependent density matrix of the (N-1)-electron system

$$\hat{\rho}^{R-DIDM}(t) = \hat{\rho}^{2+} = \mathrm{T}r_{\mu}\left[\mid \Phi^{\rho,N-1}_{m}\,(t)\,\rangle\langle\, \Phi^{\rho,N-1}_{m}\,(t) \mid\right]. \qquad (22)$$

The density matrix of the molecular dication, in the basis of ADC(2) dicationic eigenstates $\mid \Omega^{N-2,RCS}_{m,\ Probe-ionised} \rangle$, reads

$$\hat{\rho}^{2+}_{m,n}(t) = \sum_{\mu} c_{m\mu}(t)\left[c_{n\mu}(t)\right]^{*} + 2e^{i(DIP_n-DIP_m)t}\int_{-\infty}^{t}\sum_{\mu,\nu} w_{\nu,\mu} c_{m\mu}(t')\left[c_{n\nu}(t')\right]^{*} e^{-i(DIP_n-DIP_m)t'}\,dt'. \qquad (23)$$

Finally, the measurable photoemission spectrum resulting from the interaction of the x-ray probe pulse with the pump-prepared cationic system is recovered by convoluting the discrete, final populations of dicationic states $p^{2+}_{n}(t) = \left|\rho^{2+}_{n,n}(t)\right|$ with a Gaussian function of FWHM width $\delta \approx$ 2.5 eV

$$p^{Probe-pulse}_{e^-}(E) = \sum_{n} p^{2+}_{n}(\infty)\, e^{-ln(2)\left[\left(E-I^{p}_{m}-\hbar\omega_{Probe}+DIP_n\right)/2\delta\right]^{2}} \qquad (24)$$

The latter takes into account both the broadenings due to nuclear vibrations, probe pulse bandwidth and the instrument resolution. We also found that the position of the phase jump observed in the calculated photoemission spectra (see Section S2.2) is rather insensitive to changes in the parameter δ in the 1.5 – 3.5 eV range.

S2. Results.

S2.1. First ionization of the neutral species by the x-ray pump pulse: characterization of the mixed state of the cationic system prepared by pump ionization.

Pump ionization is described by directly propagating the initial ground state of the neutral molecule with the full many-body B-spline RCS-ADC(2)x/ADC(2,2) Hamiltonian. Results are shown for the equilibrium nuclear geometry of the Gly I conformer.

In the simulation we used a multicenter B-spline basis (24, 28) characterized by a radial box radius $R_{\max} = 70$, a linear grid in the center-of-mass expansion (24) with step size h = 0.3 a.u. and maximum value of the orbital angular momentum $L_{\max} = 12$. The RCS single-particle basis set we used consists of the virtual orbitals of an HF calculation performed in the cc-pVDZ GTO basis set, further truncated at the threshold energy of 2 a.u.. The number of open ionic channels included in the calculation (see Eq. (2)) and the dimension of the Hamiltonian matrix in A' symmetry space are $N_{Ionic} = 38$ and $H_{RCS-ADC}^{Maxdim} = 220500$, respectively. The values of the CAP parameters used in this calculation are $\eta = 5 \times 10^{-3}$ and $r_{CAP} = 25$ a.u. The *ab initio* simulation of the pump-step has been performed using a linearly polarized pulse, characterized by a Gaussian temporal envelope, a central frequency $\hbar\omega_{Pump} = 274$ eV, peak intensity $I_{Pump} = 6 \times 10^{15}\ \mathrm{W/cm^2}$ and time duration $T_{Pump} \sim 1$ fs (FWHM). Convergence of the results has been obtained by using a time-step $\Delta t = 0.5$ as and a Krylov-space dimension $K = 40$ in the Arnoldi/Lanczos time propagation.

The electronic state of the emerging ionic system has been fully characterized by computing the R-IDM. Fig. S1 shows the calculated degrees of quantum electronic coherence $G_{m,n}$ and the relative phases $\varphi_{m,n}$ between each pair of ionic eigenstates populated by the x-ray pump pulse. The final stationary populations $p_n(\infty)$ of the bound ADC(2,2)-calculated eigenstates of the Gly I cation are also shown in the vertical and horizontal side panels of Fig. S1. In the calculations presented here, all the ionic time-dependent populations $p_n(t)$ have converged to their final stationary value at $t \sim 200$ as after pump ionization. The final photoelectron spectrum resulting from the ionization of neutral glycine by the x-ray pump pulse is also plotted in Fig. S1. It is recovered by convoluting the calculated discrete, final populations of cationic states Gaussian function of FWHM width $\delta \approx 2.5$ eV, which takes into account both the broadenings due to nuclear vibrations, probe pulse bandwidth and the instrument resolution:

$$p_{e^-}^{Pump}(E) = \sum_n p_n(\infty)\, e^{-ln(2)\left[\left(E-\hbar\omega_{Pump}+I_n^p\right)/_{2\delta}\right]^2} \quad (25)$$

In Fig. S1, it is possible to identify islands of strong quantum electronic coherence close to the main diagonal (the highest degrees of generated quantum electronic coherence being ~ 0.95), for ionic states belonging to the 10a' (highlighted in Fig. S1) and 9a' bands. In general, a robust degree of coherence is produced between pairs of states with an energy gap up to the value of pump-pulse bandwidth, while the coherence produced between different ionic eigenstates with larger energy gaps rapidly decays to very low (< 0.2) values. The results of our *ab initio* simulation of x-ray ionization are in very good agreement with the predictions of the sudden approximation, especially in terms of the relative phases ($\varphi_{m,n}$= 0) and relative populations of the ionic states belonging to the bands which show a breakdown of the MO picture, such as the 10a' (and 9a') inner-valence ionized band for which the sudden-approximation ansatz can be written as (using Eq. (4))

$\mid \Phi^{10a',N-1}_{SUDDEN} \rangle = \mid (10a')^{-1} \rangle = \sum_m V^{+}_{10a',m} \mid \Phi^{N-1,RCS}_{m,\text{Pump-ionized}} \rangle$. The good accuracy of the sudden approximation for molecular ionization at x-ray photon energies is a result of the high (relative to the energy-scale of the bound electrons dynamics) kinetic energy of the photoelectron leaving the molecular region.

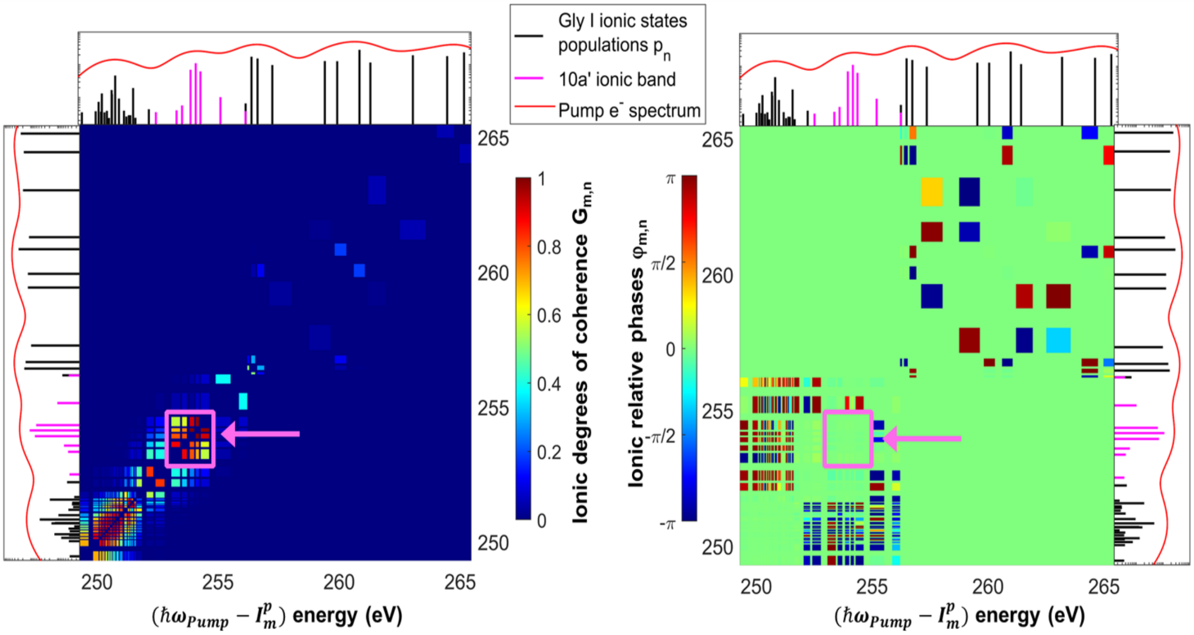

Fig. S1. Ab initio TD B-spline RCS-ADC simulation of the pump-induced first ionization of molecular glycine (Gly I conformer). The simulation is performed using the measured FLASH pulse parameters. The interaction between the pump x-ray pulse and the neutral Gly I molecule prepares a cationic system $(\text{Gly I})^+$ in a mixed state characterized by a density matrix. The left panel shows the degrees of quantum electronic coherence $\boldsymbol{G_{m,n}}$ (Eq. (9)) produced between each pair of ADC(2,2)-calculated cationic eigenstates populated by the pump pulse. The relative phases $\boldsymbol{\varphi_{m,n}}$ (Eq. (10)) between each pair of ADC(2,2)-calculated ionic eigenstates are shown in the right panel. The areas corresponding to states of the 10a' band are highlighted. The populations of the ionic eigenstates, plotted against the corresponding photoelectron energy $(\boldsymbol{\hbar\omega - I^p_m})$, are also shown in the vertical and horizontal side panels; the ionic states of the 10a' band are highlighted by a different stick colour. The kinetic energy distribution of pump-ionized electrons (Eq. (25)) is also shown in the vertical and horizontal side panels (red curve).

S2.2. Second ionization of the cationic species by the x-ray probe pulse: quantum beatings in the time-resolved photoelectron kinetic-energy distribution observable.

Results are calculated for the nuclear equilibrium geometry of the Gly I conformer. X-ray photoionization of the cationic system by the probe pulse is described here by solving Eqs. (13) and (15) for the cases of the three principal quantum-coherent pure-state channels, denoted here as $\mid \Phi^{\rho}_1 (t) \rangle$, $\mid \Phi^{\rho}_2 (t) \rangle$, $\mid \Phi^{\rho}_3 (t) \rangle$, as obtained in Eq. (11) by the purification of the ionic density matrix prepared by the pump pulse. These principal, coherent ionic channels correspond to the coherent superpositions of ionic states in the 10a' ($I^p_{10a'} \sim 20$ eV, $r_1 = 0.15$) and 9a' ($I^p_{9a'} \sim$

23 eV, $r_3 = 0.09$) bands, respectively, as well as the coherent superposition of the two ionic states consisting of 11a' and 12a' hole-mixing ($r_2 = 0.13$) at an energy $I^p_{11a'/12a'} \sim 17.5$ eV.

The absolute value of the expansion coefficients of these pure-state channels (in the basis of the 38 ionic eigenstates $\{| \, \Phi^{N-1,RCS}_{m,\text{Pump-ionized}} \rangle\}$ included in the simulation of the x-ray pump ionization step) as calculated by Eq. (11), are shown in the insets of Fig. S3. The other pure-state channels contributing to the expansion of Eq. (11) are characterized by a negligible quantum coherence in the basis of the cationic eigenstates and therefore do not contribute to the observed oscillations in the photoelectron observables.

In order to illustrate the charge dynamics triggered by the x-ray pump ionization, in Fig. S2 we show the hole densities (defined as the difference between the electron density of the neutral ground state and the one of the pump-prepared correlated ionic state), corresponding to the 10a' band coherent ionic channel $| \, \Phi^{\rho}_1 \, (t) \, \rangle$, at ten different times, namely immediately after the pump ionization event at t_1 = 1 fs up to t_{10} = 21.4 fs after it covering one full charge oscillation period.

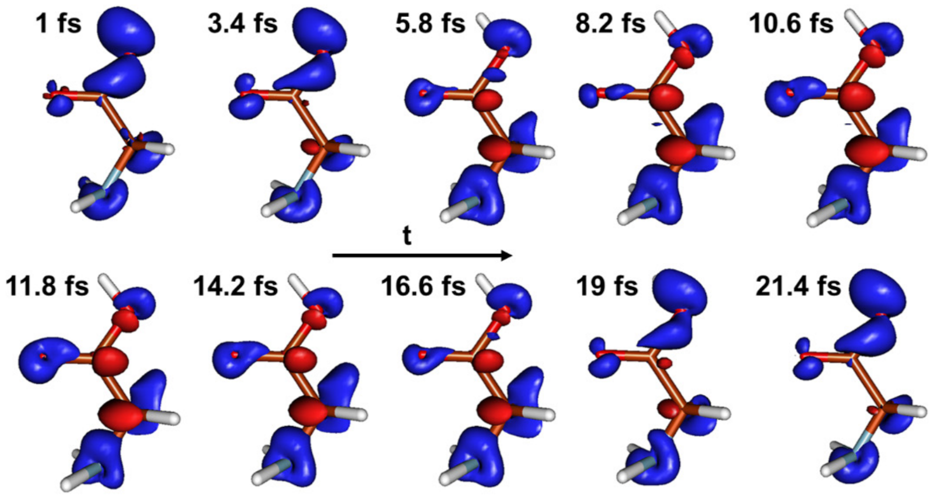

Fig. S2. Hole densities corresponding to the correlated 10a' pure state channel between t_1 = 1 fs and t_{10} = 21.4 fs after the pump ionization event. The density iso-surfaces displayed are the ones with value 0.015, blue and red colors indicate positive and negative values of the hole density, respectively.

The time propagation is performed by means of the Arnoldi/Lanczos algorithm. During the simulation of the probe step, we have included in the expansion of the many-electron wavefunction Eq. (15) all the open doubly-ionized channels with energy up to 3.5 a.u. The resulting number of open dicationic channels included in the simulation is $N_{Dication} = 225$. The highest value (among the different molecular symmetry spaces) of the Hamiltonian matrix dimension is $H^{Maxdim}_{RCS-ADC} =$ 1335318. The other numerical parameters are the same as for the simulation of the pump step (see Section S2.1).

To reduce the demanding computational effort resulting from the size of the Hamiltonian matrix, we employed the following approximation and neglected the interchannel couplings in the continuum between different dicationic channels, i.e. we set

$\langle \, \Phi^{N-1}_{\nu,n'} \, | \, \hat{H}^{N-1}_{RCS-ADC} \, | \, \Phi^{N-1}_{\mu,n} \rangle \; = \; 0, \;\; n' \; \neq \; n.$

Fig. S3 shows the relative variation, as a function of the x-ray pump-probe time-delay τ_d, of the separate contributions to the probe-induced electron yield $p_{e^-}^{Probe-pulse}$ (Eq. (24)) from the three pure-state ionic coherent superpositions associated with the 10a', 11a'/12a' and 9a' bands, respectively. The change of electron yield is plotted relative to the time-delay averaged value. Since the time-delay oscillation of the relative change in $p_{e^-}^{Probe-pulse}(E,\tau_d)$ shows, for each coherent ionic channel, a change of phase by π in different regions of the kinetic energy spectrum, we plot the integrated $p_{e^-}^{Probe-pulse}(\tau_d)$ yield in both the kinetic energy ranges above and below the phase jump position resulting from the *ab initio* numerical calculations.

The π difference observed in the experiment between the phase of oscillation of the photoelectron signal in the lower and higher kinetic energy ranges of the photoelectron spectrum, respectively, is due to the different time evolution of the 1h and 2h1p components of the time-dependent ionic state created by the pump pulse in the 10a' band. The latter can in fact be written as a time-dependent linear combination of the 10a' 1h configuration (hole in the 10a' orbital) and several 2h1p configurations, which can, in principle, have holes in any of the outer-valence and inner-valence molecular orbitals. As far as the dicationic eigenstates are concerned, they can be approximated as linear combinations of 2h and three-hole, one-particle (3h1p) configurations. From energy considerations, it appears clear that configurations with one hole in the 10a' orbital will mostly contribute to highly-excited dicationic eigenstates, while low-energy dicationic eigenstates will mostly consist of two holes in outer-valence molecular orbitals. Moreover, it is also clear that ionization into highly-excited dicationic states will be accompanied by the emission of photoelectron within the lower kinetic energy range, compared to the ones accompanying ionization into the lowest excited dicationic states. According to the sudden approximation, whose accuracy we have verified to be high in the photon energy range of the experiment, immediately after ionization by the pump pulse the linear combination is such that the only (time-dependent) coefficient appreciably different from zero is the one of the 10a' 1h configuration.

Therefore, on the one hand, as the pump-probe time-delay increases, the evolution of the time-dependent ionic state is such that the amplitude of the 10a' 1h coefficient decreases. As a result, since most of the oscillator strength to highly-excited dicationic states with (at least) one hole in the 10a' orbital is carried by this part of the time-dependent state, also the photoelectron signal in the low kinetic energy range initially decreases. On the other hand, the amplitude of the 2h1p coefficients initially increases. Since these carry most of the oscillator strength for ionization transitions to the lowest-energy dicationic states, the higher-kinetic energy photoelectron signal will also initially increase. This explains the π phase difference between different kinetic energy regions of the photoelectron spectrum.

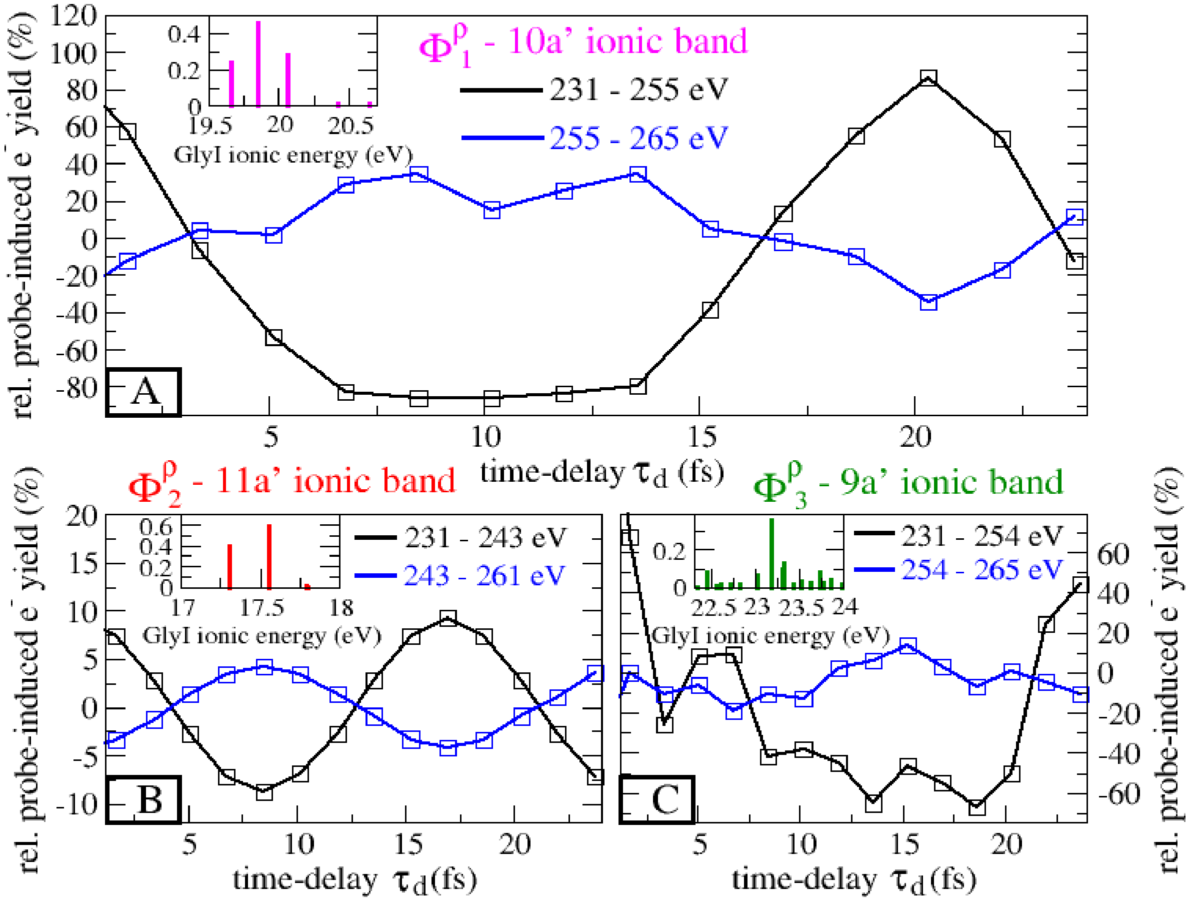

Fig. S3. Ab initio TD B-spline RCS-ADC simulation of the interaction between the probe pulse and the pump-prepared cationic system (Gly I conformer). The simulation is performed using the measured FLASH pulse parameters. (A) Relative change of the probe-produced electron yield in the kinetic energy ranges above and below the phase jump, as a function of x-ray pump-probe delay; contribution from the pump-prepared pure-state ionic channel consisting of a coherent superposition of eigenstates in the 10a' ionic band. In both energy ranges the electron yield includes contributions from Auger emission and sequential double ionization as a function of x-ray pump-probe delay. **(B)** and **(C)** – same as **(A)** for the contribution of the pump-prepared pure-state ionic channels consisting of coherent superpositions of eigenstates in the 11a'/12a' and 9a' ionic bands, respectively.

The calculated position of the phase jump for the 10a' and 9a' coherent ionic channels (~255 eV) appears shifted to a higher value of the electron kinetic energy with respect to the one observed in the experimental data. This deviation shows that, despite the excellent agreement in capturing at a qualitative level the phase-jump mechanism, the theoretical modeling of the final doubly-ionized states $|\,\Omega^{N-2,RCS}_{n,Probe-ionised}\rangle$ adopted here does not accurately include the effect of electron correlation in the populated final states of the glycine dication. The part of the spectrum where the phase jump is observed in the experiment is a region of complete breakdown of the MO picture of double ionization, characterized by a high density of doubly-ionized satellite states. As a result, the contribution of 3h1p configurations to the description of the states in this energy region is very

strong. Since this contribution is not included here (at the ADC(1) level, 2h configuration mixing is taken into account), the absolute position of the dicationic energy band is not well-captured at a quantitative level, leading to a shift of the predicted phase-jump position.

In both energy ranges the electron yield includes contributions from both the direct sequential double ionization (SDI) by the probe pulse of the cationic states populated coherently by the pump pulse, and the Auger emission from the probe-excited 4a' and 5a' core-ionized Auger channels.

In order to quantify the relative contribution of these two mechanisms, in Fig. S4 we show the relative change, as a function of the x-ray pump-probe time-delay, of the total contribution from the pump-prepared cationic mixed state to probe-induced electron yield in the kinetic energy ranges above and below the phase jump. For comparison, we also plot the isolated contribution due to electrons emitted through Auger-decay. The total spectrum shown in Fig. S4 consists of the incoherent sum of the individual contributions from the 3 coherent pure-state channels shown in Fig. S3. The Auger electron contribution for each coherent channel is calculated as

$$p_{e^-}{}^{Auger}(E) = \sum_n p_n^{2+,Auger}(\infty)\, e^{-ln(2)\left[\left(E-I_m^p-\hbar\omega_{Probe}+DIP_n\right)/_{2\delta}\right]^2} \qquad (26)$$

by integrating the CAP term of the diagonal dicationic density matrix (Eq. (23)) starting from $t = 0.5$ fs after the probe pulse

$$p_n^{2+,Auger}(\infty) = \left|\rho_{n,n}^{2+,Auger}(\infty)\right| = \left|\lim_{t\to\infty} 2\int_{0.5fs}^{t} \sum_{\mu,\nu} w_{\nu,\mu} c_{m\mu}(t')[c_{n\nu}(t')]^*\, dt'\right| \qquad (27)$$

The validity of this formula is based on the different time-scales corresponding to direct and Auger electron emission, and by the fact that the photoelectron wave-packet emitted as a result of direct photoionization by the probe pulse is completely absorbed by the CAP within the first 0.3 fs after the probe pulse. The total Auger electron contribution presented in Fig. S4 thus describes the total yield of electrons emitted in the Auger decay of the C(1s) 4a' and 5a' core-ionized states populated by the probe pulse.

The result of Fig. S4 show that, at the photon energy value used in the present experiment, the direct photoionization dominates over the excitation of the intermediate Auger-active core-singly-ionized C(1s) resonances. The calculated Auger signal is indeed about one order of magnitude smaller than the SDI one.

Therefore, the quantum-coherent electron dynamics prepared by the x-ray pump in the cation is imprinted in the direct probe-ionized electron signal, which dominates over the Auger-induced electron signal. Moreover, the results show that the photoelectron signal is more sensitive to the quantum coherence in the 10a’ band compared to the coherences produced in the 11a’/12a’ and 9a’ ionic bands: this is because the pure-state ionic channel consisting of a coherent superposition of eigenstates in the 10a’ band is characterized by a higher population weight $r_{10a'} = 0.15$ (see Schmidt decomposition in Eq.(11)), and, as shown in Fig. S3, also gives rise to higher-amplitudes in the calculated oscillation of the electron yield. As a consequence, the position of the phase jump in the total time-delay dependent electron yield is also approximately the same as the one of the 10a’ coherent channel alone.

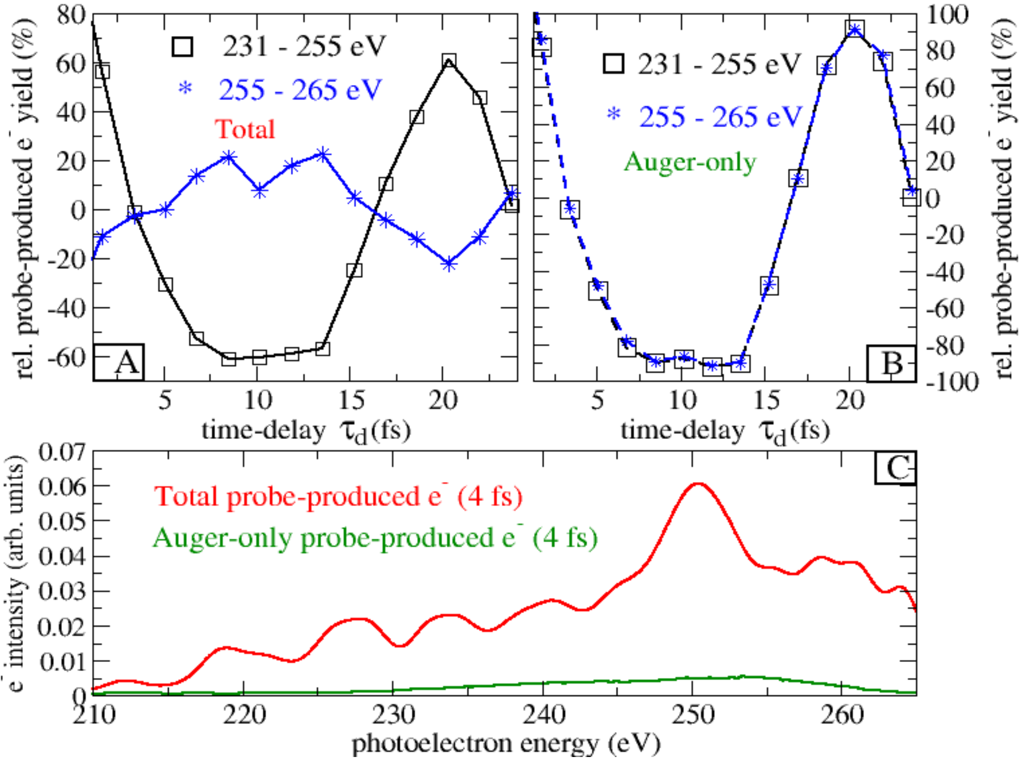

Fig. S4. Ab initio TD B-spline RCS-ADC simulation of the interaction between the probe pulse and the pump-prepared cationic system (Gly I conformer). The simulation is performed using the measured FLASH pulse parameters. (A) Relative change of the probe-produced electron yield in the kinetic energy ranges above and below the phase jump, as a function of x-ray pump-probe delay; total contribution from the pump-prepared mixed-state of the ionic system. In both energy ranges the electron yield includes contributions from Auger emission and sequential double ionization as a function of x-ray pump-probe delay. **(B)** same as **(A)** for the isolated contribution of Auger electrons, resulting from the decay of the 5a' and 4a' core-ionized states populated by the probe. **(C)** Kinetic energy distributions of the probe-induced Auger electrons (green curve) and the complete (Auger + SDI) probe-emitted electrons (red curve); the latter includes the dominant contribution of the photoelectron emitted in the process of (second) direct photoionization of the cationic system by the probe pulse.

S3. Effect of nuclear ground-state geometry distribution: time-dependent survival probability of the pump-prepared mixed state.

Here we analyze the effect of the geometry-spread of the ground state nuclear wavefunction on the survival of the quantum electronic coherences in the 10a' and 9a' ionized bands. The simulations were performed for and averaged over 201 different nuclear geometries, at which convergence of the calculated ionic density matrix survival probability was found.

S3.1. Numerical procedure.

The entire procedure of the calculation is outlined below. For each and every nuclear geometry, the calculation we performed consists of three elements:

1- Calculation of the bound ionic states of glycine, in the 10a' and 9a' bands energy regions. This calculation was performed at the advanced ADC(2,2) level of theory (27) by using the cc-pVDZ basis set with the virtual orbital space truncated at the threshold energy of 2 a.u. Here, only ionic eigenstates with more than 1% 10a' or 9a' contribution in their full ADC configuration expansion are kept.

2- The pump-prepared initial mixed-state of the cationic system, characterized by the ionic density matrix $\hat{\rho}(t_0)$, is time-propagated by solving the (field-free) time-dependent von Neumann equation (26)

$$\frac{d}{dt}\hat{\rho}(t) = -\frac{i}{\hbar}\left[\hat{H}_{ADC(2,2)}^{N-1,RCS}, \hat{\rho}(t)\right], \quad (28)$$

$$\hat{\rho}(t) = e^{-\frac{i}{\hbar}\hat{H}_{ADC(2,2)}^{N-1,RCS}(t-t_0)}\,\hat{\rho}(t_0)\,\exp^{+\frac{i}{\hbar}\hat{H}_{ADC(2,2)}^{N-1,RCS}(t-t_0)}. \quad (29)$$

For each nuclear geometry, the initial $\hat{\rho}(t_0)$ reduced ionic density matrix is built according to the following criteria, which we name *coherence-corrected sudden-approximation ansatz*:

- The relative populations of the valence ionic eigenstates (both in the 10a' and 9a' bands), as well as the relative phases between each pair of eigenstates, are estimated using the sudden approximation ansatz to model the ionization of neutral glycine by the pump pulse. Therefore, each ionic eigenstate has a population proportional to the weight of the simple one-hole configurations in their ADC configuration expansion, and the relative phases are 0.
- We assume a degree of quantum coherence different from zero only between ionic eigenstates of the same symmetry, and consequently do not include ionic states of a'' symmetry in our description. Assuming a coherence different from zero only between ionic states of the same symmetry implies that the direction of the first emitted photoelectron is either not measured or integrated out in the analysis of the experimental data. At a theoretical level, it corresponds to calculating the ionic density matrix by tracing the full N-electron neutral wavefunction over the spatial-symmetry quantum numbers of the photoelectron.
- Finally, for each pair of coherently populated ionic eigenstates, the degree of coherence is further decreased (from the initial value of 1) in order to take into account the effect of their energy separation, the bandwidth and profile of the pump pulse. Therefore, the off-diagonal matrix elements $\rho_{m,n}(t_0)$of the ionic density matrix are modified according to

$$\rho_{m,n}(t_0) = \rho_{m,n}^{Sudden}\, e^{-\ln(2)\left(\frac{E_m - E_n}{E_{Band}}\right)^2}, \tag{30}$$

which provides a reliable estimate of the initial ionic density matrix prepared as a result of ionization by a 274 eV x-ray Gaussian pump pulse of bandwidth E_{Band}.

The validity of this ansatz is confirmed, at the nuclear equilibrium geometry, by the *ab initio* results of Fig. S1.

3- Given the density matrix $\hat{\rho}(t)$, the survival probability of the time-dependent quantum state is calculated as the fidelity between the quantum states described by the density matrix $\hat{\rho}(t)$ and the initial density matrix prepared by the pump $\hat{\rho}(t_0)$

$$\mathrm{F}(t) = \left(Tr\left[\sqrt{\sqrt{\hat{\rho}(t)}\,\hat{\rho}(t_0)\sqrt{\hat{\rho}(t)}}\right]\right)^2. \tag{31}$$

S3.2. Nuclear geometry sampling.

The optimization of the equilibrium geometry $\{\overrightarrow{\boldsymbol{R_n}}\}_0$ and the calculation of the normal-mode frequencies were computed at the coupled-cluster singles and doubles (CCSD) level of theory in a cc-pVTZ basis set, using the MOLPRO quantum chemistry package (30); the different nuclear geometries were sampled in the configuration space according to a probability distribution given by the Wigner distribution, integrated over momenta, corresponding to the nuclear ground-state (GS) wavefunction, i.e. $\left|\Lambda_{GS}^{Nuclear}\left(\{\overrightarrow{\boldsymbol{R_n}}\}\right)\right|^2$. The latter was calculated within the harmonic oscillator approximation (around the equilibrium geometry) of the potential energy surfaces. Therefore, the ensemble of geometries was calculated using a product of normal Gaussian distributions, each one corresponding to the respective vibrational normal mode considered. Only vibrational normal modes that conserve the molecular symmetry of the equilibrium geometry (C_s point group) were considered. Monte Carlo integration of the time-dependent survival probability curves over different nuclear geometries $\{\overrightarrow{\boldsymbol{R_n}}\}$ was performed. The aforementioned procedure describes the effect of the spread of the GS nuclear wavefunction on the observed coherent electron dynamics. Vibrational dynamics is not included in the simulations.

S3.3. Results.

Fig. S5 shows the geometry-averaged, time-delay-dependent survival probability of the ionic density matrix prepared by the x-ray pump ionization. Results are presented for the two conformers Gly I and Gly III, as well as for three different statistical mixings of the populations of the two conformers in the sample.

The results for both the singly-ionized Gly I conformer and the averaged abundance-weighted contribution of the two (Gly I and Gly III) conformers, show that it is possible to discern an oscillation with a ~ 20 fs period in the time-dependent survival probability of the cationic mixed state prepared by the pump ionization. The overall agreement with the experimental results is excellent in terms of the oscillation period and it provides strong evidence in support of the presence of an electronic coherence-driven oscillation characterized by a period of ~ 20 fs and whose coherence partially survives the nuclear GS distribution averaging.

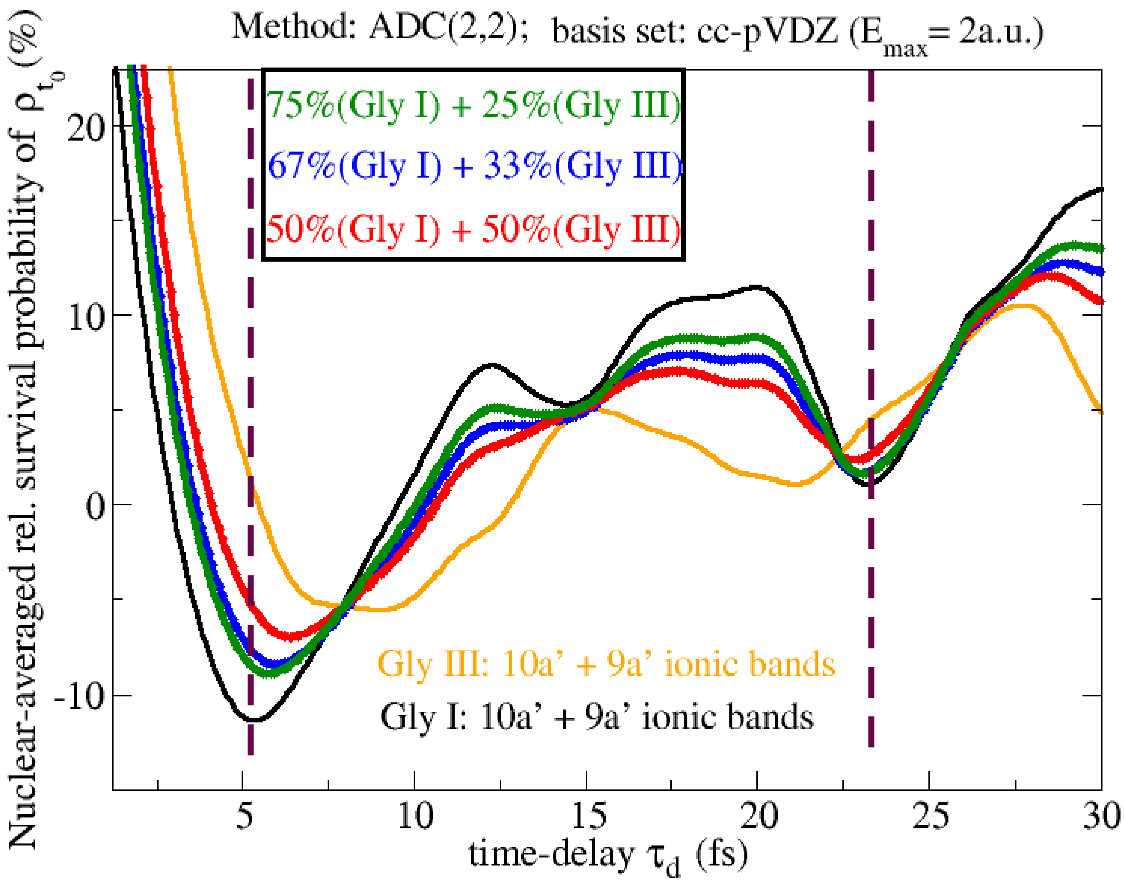

Fig. S5. Time-dependent survival probability of the pump-prepared ionic density matrix in the subspace of ionic eigenstates consisting of the 10a' and 9a' ionic bands. The result of Eq. (31) has been averaged over the nuclear ground-state geometry distribution for both the Gly I and Gly III conformers, as well as for three different statistical mixtures of the two.

Here it is important to note that, whereas both the time-dependence and the temporal resolution of the measured physical observable does in principle present deviations from the calculated survival probability, the robustness of the latter with respect to the nuclear geometry averaging provides a probe-free demonstration of the survival of quantum electronic coherence on the tens-of-femtosecond time-scale in this system.

Regarding the profile of the oscillation, neither the theory results nor the bare experimental data points show a simple, perfectly singled-period oscillation. Moreover, the amplitude of the calculated oscillation is around 10% (measured against the time-delay averaged value), and therefore smaller than the experimentally measured one which ranges from 15% up to 30%.

The observed difference in the relative amplitude of the oscillations has to be considered remarkably small, and thus supportive of the presented experiment's interpretation, especially considering the potential theoretical sources of discrepancy (in addition to the experimental errors), which include:

- Errors due to the use of a truncated single-particle basis set in the calculation of the 10a' and 9a' ionized states at the ADC(2,2) level of the ADC hierarchy. This can in principle have an appreciable impact on the observed discrepancy, as it can lead to relative errors in the excited ionic states expansions and consequently in the energy gaps between different ionic eigenstates as well as in the geometry of the corresponding potential energy surfaces.
- Deviations, as a function of the nuclear geometry, of the relative ionic populations and ionic coherences, estimated by means of the procedure outlined above, with respect to the pump-prepared ones. The vertical ionization probabilities by the pump could potentially present deviations from the theoretically estimated one for the states involved, depending on the vertical ionization energies at different geometries.
- The effect of phenomena not-included in the present analysis:
    i. the symmetry-breaking vibrational normal modes.

    the ionization-induced nuclear motion.

    ii. Given the relatively high temperature of the molecular sample used in the experiment, contributions from excited vibrational states could also play a non-negligible role.

Movie S1.

The movie depicts the calculated temporal evolution of the relative variation of the hole density corresponding to the correlated 10a' pure state channel between 1 fs $< t <$ 21.4 fs after the pump ionization event with respect to its time-averaged value. The density iso-surfaces displayed are the ones with value 0.015, blue and red colors indicate positive and negative values of the hole density, respectively.
