# Supplementary figures and images for: Electronic quantum coherence in glycine molecules probed with ultrashort x-ray pulses in real time
Source: Sci Adv. 2022 Jun 1;8(22):eabn6848. doi: 10.1126/sciadv.abn6848 (PMC9159702; doi:10.1126/sciadv.abn6848)

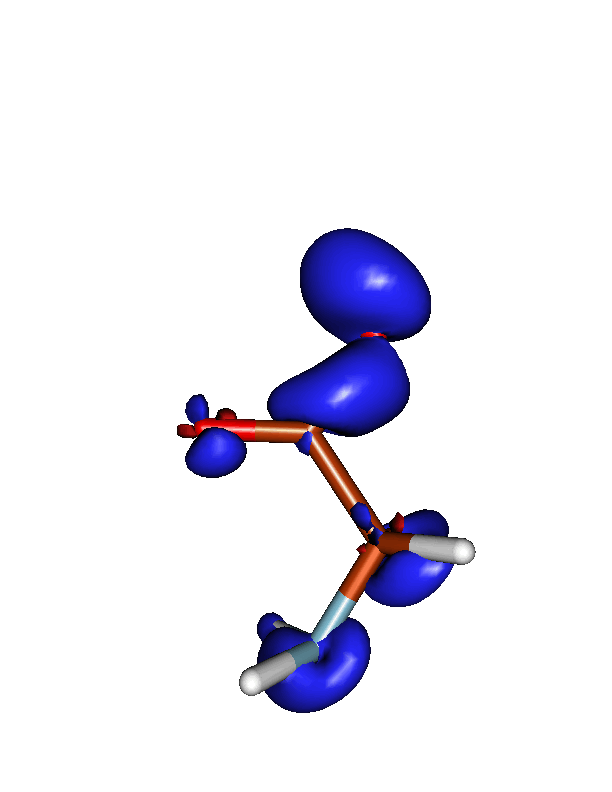

Supplement: Supplementary file 2 — Movie S1 [file sciadv.abn6848_movie_s1.zip › sciadv.abn6848_movie_s1.gif]
